# Comparison of pharmacokinetic profiles of seven major bioactive components in normal and non-alcoholic fatty liver disease (NAFLD) rats after oral administration of Ling-Gui-Zhu-Gan decoction by UPLC-MS/MS

**DOI:** 10.3389/fphar.2023.1174742

**Published:** 2023-05-04

**Authors:** Wenlong Nie, Yang Yang, Ling Li, Yue Ding, Xingmi Chen, Ming Li, Ning He, Guang Ji, Yong Zhang, Ping Kang, Tong Zhang

**Affiliations:** ^1^ School of Pharmacy, Shanghai University of Traditional Chinese Medicine, Shanghai, China; ^2^ Experiment Center for Science and Technology, Shanghai University of Traditional Chinese Medicine, Shanghai, China; ^3^ School of Pharmacy, Anhui University of Chinese Medicine, Hefei, Anhui, China; ^4^ Experiment Center for Teaching and Learning, Shanghai University of Traditional Chinese Medicine, Shanghai, China; ^5^ Institute of Digestive Diseases, Longhua Hospital, Shanghai University of Traditional Chinese Medicine, Shanghai, China

**Keywords:** NAFLD, UPLC-MS/MS, comparative pharmacokinetics, rat plasma, Ling-Gui-Zhu-Gan decoction

## Abstract

A sensitive and rapid ultra-performance liquid chromatography–tandem mass spectrometry (UPLC-MS/MS) method was hereby developed for the determination of seven components, namely, glycyrrhizic acid, glycyrrhetinic acid, dehydrotumulosic acid, isoliquiritin, liquiritin, atractylenolide III, and cinnamic acid, in the plasma of rats after the oral administration of Ling-Gui-Zhu-Gan decoction (LGZGD). Besides, this very method was methodologically validated for specificity, linearity, inter-day and intra-day precision, accuracy, matrix effect, extraction recovery, and stability. It was also successfully used for the first time to compare the pharmacokinetic characteristics of the seven components after oral administration of LGZGD to normal rats and non-alcoholic fatty liver disease (NAFLD) rats. The results indicated significant differences between the pharmacokinetic characteristics of normal and NAFLD rats. To further reveal the different pharmacokinetic behaviors, the expressions of enzymes and transporters in the liver of normal and NAFLD rats were detected using UPLC-MS/MS. In the NAFLD rats, UDP-glucuronosyltransferase 1-1 (UGT1A1) and nine transporters were significantly inhibited and a positive correlation was observed between them and the AUC of the major components. The present results indicate that the pharmacokinetic differences between the normal and NAFLD rats might be attributed to the significant lower expression levels of both the metabolic enzyme UGT1A1 and nine transporter proteins in the NAFLD rats than in the normal rats. Meanwhile, UGT1A1 and the nine transporter proteins might be used as potential biomarkers to assess the ameliorative effect of LGZGD on NAFLD, which could provide useful information to guide the clinical application of LGZGD.

## 1 Introduction

Non-alcoholic fatty liver disease (NAFLD) is a common chronic liver disease featuring an increasing incidence year after year. The lack of safe and effective drugs for thorough treatment has made related cardiovascular diseases a major problem of chronic diseases (Lou et al., 2020; Gu et al., 2021). To this end, the PubMed/MEDLINE literature covering the epidemiology and progress of NAFLD from 1989 to 2015 has hereby been searched, and the global prevalence of NAFLD estimated to be 25.24% (95% confidence interval: 22.10–28.65) (Younossi et al., 2016). The prevalence is the highest in the Middle East and South America and the lowest in Africa. As the global obesity epidemic intensifies, the clinical and economic burden of NAFLD becomes enormous (Zhou et al., 2019). Given that people with non-alcoholic steatohepatitis are exposed to more severe liver damage than those with steatosis alone, non-alcoholic steatohepatitis leads to further fibrosis of the liver, ultimately leading to liver-related illness and death (Diehl et al., 2017), making it necessarily important to actively prevent and treat NAFLD. However, there is currently no ideal treatment for NAFLD. Although traditional Chinese medicine has become a common treatment for NAFLD in China and its efficacy, specificity, and advantages are gradually being confirmed; however, its specific mechanism still requires further exploration (Li and Hu, 2020). Ling-Gui-Zhu-Gan decoction (LGZGD) is derived from *Treatise on Febrile Diseases* and *Synopsis of the Golden Chamber* drafted by Zhongjing Zhang in the Han dynasty (Commission and C. P., 2020), which is made of four herbs, namely, *Poria cocos* (Schw.) Wolf (Fuling), *Cinnamomum cassia* Presl (Guizhi), *Atractylodes macrocephala* Koidz. (Baizhu), and *Glycyrrhizae Radix et Rhizoma* (Gancao), in the ratio 4:3:3:2 (w/w/w/w) (Gu et al., 2020). LGZGD features an extensive clinical application and a favorable clinical effect. In clinical practice, LGZGD is generally used to treat cardiovascular diseases (Song et al., 2003). In recent years, with the increasing popularity of LGZGD in the treatment of different diseases, such as mental disease, type 2 diabetes, hypertension, and heart failure, the novel applications of LGZGD are constantly discovered (Ding et al., 2005; Chen et al., 2011; Wang and Xiong, 2012; Wang et al., 2020a; Wang et al., 2020b). LGZGD has also been shown to alleviate hepatic steatosis caused by high-fat diets. Yang et al. and Gu et al. found that LGZGD can promote the metabolism of fat and is thus used to prevent and cure NAFLD (Yang et al., 2012; Gu et al., 2020). Besides, LGZGD has also been found capable of regulating the Nrf2/ARE signaling pathway to improve NAFLD in rats (Guo et al., 2017), which provides a theoretical basis for the clinical treatment of NAFLD.

**FIGURE 1:**
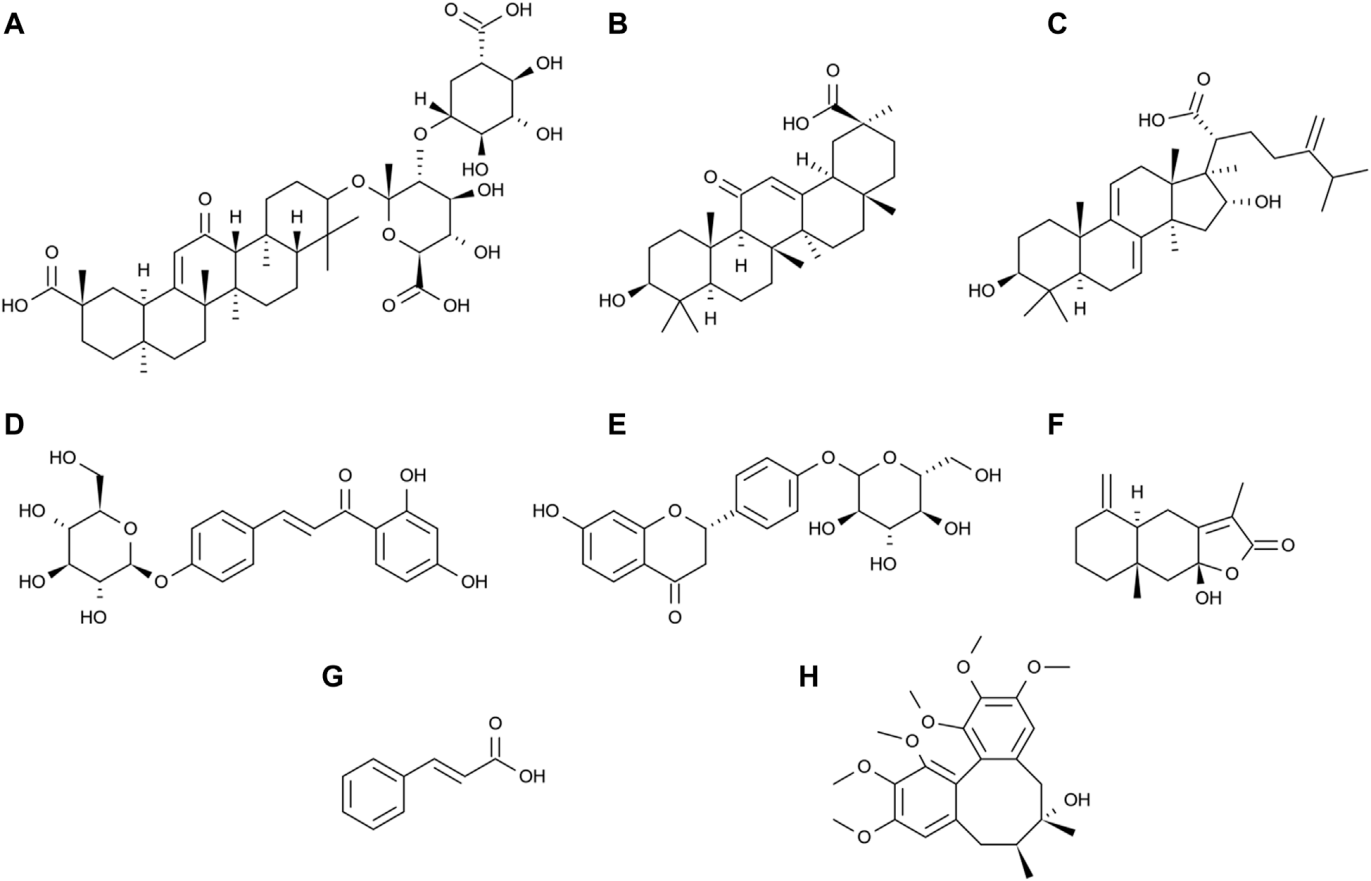
Chemical structures of glycyrrhizic acid **(A)**, glycyrrhetinic acid **(B)**, dehydrotumulosic acid **(C)**, isoliquiritin **(D)**, liquiritin **(E)**, atractylenolide III **(F)**, cinnamic acid **(G)**, and schisandrin (IS) **(H)**.

Pharmacokinetic studies of drug absorption components can help in understanding the toxicity, safety, and *in vivo* processes of drugs. Previous studies on LGZGD have focused on phytochemistry and pharmacology, but pharmacokinetic studies of LGZGD components in rats have been rarely reported. Pharmacological studies have shown that the main active compounds in *Glycyrrhizae Radix et Rhizoma* include glycyrrhizic acid (Figure 1A), glycyrrhetinic acid (Figure 1B), isoliquiritin (Figure 1D), and liquiritin (Figure 1E). Glycyrrhetinic acid exerts anti-NAFLD effects by regulating the intestinal microbiota. It has been revealed that the function of carbohydrate transport and metabolism is significantly decreased by glycyrrhizic acid. Glycyrrhetinic acid prevents cholestatic liver injury in bile duct–ligated rats, while liquiritin alleviates cyclophosphamide-induced liver sinusoidal endothelial injury and inflammatory injury in mice (Chen M. et al., 2019; Pan et al., 2022; Wang et al., 2022). *Poria cocos* (Schw.) Wolf extracts protect the liver from acute ethanol insult (Yimam et al., 2016). The main active compound in *Poria cocos* (Schw.) Wolf is dehydrotumulosic acid (Figure 1C), which has anti-inflammatory, antitumor, antiviral, and immuno-promoting effects (Ukiya et al., 2002; Akihisa et al., 2007; Xiao et al., 2012). Besides, the main active compound in *Atractylodes macrocephala* Koidz is atractylenolide III (Figure 1F), which has been reported to ameliorate NAFLD by activating the hepatic adiponectin receptor 1–mediated AMPK pathway. After administration, it can significantly reduce the serum levels of alanine aminotransferase and glutamic oxaloacetic transaminase in mice, and alleviate hepatic oxidative stress (Li et al., 2021). Meanwhile, the main active compound in *Cinnamomum cassia* Presl is cinnamic acid (Figure 1G), which can ameliorate NAFLD by suppressing hepatic lipogenesis and promoting fatty acid oxidation (Wu et al., 2021). A high-performance liquid chromatography method for the determination of cinnamic acid and dehydrotumulosic acid in rat plasma was thus developed (Song et al., 2002; Song et al., 2003). The rat plasma samples were acidified with hydrochloric acid and extracted with ethyl acetate. The established method is found to be sensitive and specific, but not fast enough for the rapid determination of a large number of biological samples. The lower limits of quantification (LLOQ) of cinnamic acid and dehydrotumulosic acid by high-performance liquid chromatography are 840 and 200 ng/mL, respectively, which are considered not clinically sensitive. Additionally, the ultraperformance liquid chromatography–tandem mass spectrometry (UPLC-MS/MS) method has been reported to be used in studying the plasma samples of oral LGZGD in rats, and 11 compounds have been successfully analyzed, but the determination time was relatively long (Ji et al., 2018). Therefore, a rapid and sensitive UPLC-MS/MS method is required to determine the components in the plasma after the oral administration of LGZGD.

The pharmacokinetic drug behavior is different in normal and NAFLD rats. The pharmacokinetics of rosiglitazone in normal and high-fat diet–induced NAFLD model rats have hereby been reported (Kulkarni et al., 2016). The results have shown that the pharmacokinetics of rosiglitazone in NAFLD mice changed significantly. Compared with healthy mice, the oral clearance rate of rosiglitazone in NAFLD mice was significantly reduced and the average plasma half-life was significantly prolonged. Actually, NAFLD has been reported to be associated with the regulation of metabolic enzymes and transporters (Fisher et al., 2009). The difference in the pharmacokinetics might be related to changes in the expression of transporters in rats. Therefore, studying the changes of metabolic enzymes and transporters in rat liver is of great significance to reveal the pharmacokinetic process in NAFLD rats. On the basis of the previous research by the present researchers (Yi et al., 2018; Ma et al., 2021), UPLC-MS/MS was used to detect the expression levels of metabolic enzyme UGT1A1 and drug transporters NTCP, OATP1A1, OCT1, MATE1, MRP2, MDR1, BSEP, OATP1A2, and OATP1A4 in the liver of rats in the normal and model groups. Up to now, no comparative pharmacokinetics of these active compounds has been reported in normal and NAFLD rats after LGZGD administration. Therefore, an accurate and reliable UPLC-MS/MS method is hereby proposed for the simultaneous determination of seven components in the plasma of normal and NAFLD rats after LGZGD administration. The results showed significant differences in pharmacokinetic characteristics between the normal and NAFLD groups. The method was fully validated and applied in a comparative pharmacokinetic study. Overall, the results can provide useful information for clinical application.

## 2 Materials and methods

### 2.1 Chemicals and reagents

The standards which included glycyrrhizic acid, glycyrrhetinic acid, dehydrotumulosic acid, isoliquiritin, liquiritin, atractylenolide III, cinnamic acid, and schisandrin (≥98% purity) were all procured from Hongyong Biotechnology Co., Ltd. (Shanghai, China). Sulfatase was procured from Helix pomatia (Type H-1, sulfatase ≥10, 000 units/g solid, Sigma, United States). The peptides LTIIPQDPILFSGSLR, GVALPETIEEAENLGR, AAATEDATPAALEK, TFQFPGDIESSK, LLLSGFQEELR, STALQLIQR, NTTGALTTR, EENLGITK, SVQPELK, and TYPVPFQR, as well as stable isotope-labeled internal standards (≥98% purity) were synthesized by Bankpeptide Biological Technology, Co., Ltd. (Hefei, China). The ProteoExtract Native Membrane Protein Extraction Kit was purchased from Merck KGaA (Darmstadt, Germany). The BCA Protein Assay Kit and In-Solution Tryptic Digestion Kit were obtained from Pierce Biotechnology (Rockford, IL, United States). Formic acid dedicated to mass spectrometry (99% purity); ammonium bicarbonate (98% purity); and sodium deoxycholate (98% purity) were purchased from Sinopharm Chemical Reagent Co., Ltd. (Shanghai, China). Acetonitrile and methanol, both MS grades, were obtained from Merck KGaA (Darmstadt, Germany).

### 2.2 Preparation of LGZGD extract

LGZGD, consisting of Fuling: 120 g, Guizhi: 90 g, Baizhu: 90 g, and Gancao: 60 g, was soaked in 2,880 mL of deionized water (1:8, w/v) for 0.5 h and reflux extracted for 1.5 h. The right amount of aromatic water was collected for the first time. Then, 2,880 mL of deionized water was added for reflux extraction for another 1.5 h. The extract solutions were mixed and concentrated to an appropriate amount, and LGZGD extract powder was obtained by spray drying. The inlet air temperature of the spray dryer was controlled at 180°C ± 5°C; the outlet air temperature was 90°C–100°C; the dry paste moisture was below 5%; the atomization frequency was 200 Hz; and the extract yield was 17.56%. The aromatic water was taken and β-cyclodextrin inclusion was added to it, and the inclusion compound was mixed with the extract powder. The content of the analyte in the LGZGD extract was determined by the UPLC-MS/MS method. The amount of glycyrrhizic acid, glycyrrhetinic acid, dehydrotumulosic acid, isoliquiritin, liquiritin, atractylenolide III, and cinnamic acid in the content was 5360.3, 16.4, 6.0, 290.5, 6991.5, 141.8, and 739.2 μg/g, respectively.

### 2.3 Animals handing

Male Sprague–Dawley rats, weighing 200–220 g, SPF grade, were purchased from Shanghai Sippr-BK laboratory animal Co. Ltd. (Shanghai, China) and raised in the Laboratory Animal Center of Shanghai University of Traditional Chinese Medicine. The rats were kept in an environmentally controlled condition: on a 12:12 h light–dark cycle (lights on at 7:00 a.m.) with regulated temperature and humidity, at a temperature of 22°C–24°C and a relative humidity of 60–65%. In the experiment, the rats in the normal group were fed a certified standard rat diet and tap water, while those in the model group were fed a high-fat diet for 8 weeks to construct the NAFLD model (high-fat diet: 2% cholesterol, 10% lard, 0.1% sodium cholate, 10% yolk powder, and 77.9% normal diet). The animal experiment program was approved by the Animal Committee of Shanghai University of Traditional Chinese Medicine (License No.: PZSHUTCM201016001).

### 2.4 Pharmacokinetic study of LGZGD in normal and NAFLD rat models

#### 2.4.1 Instrumentation and chromatographic conditions

An Agilent 1290 UPLC system was combined with an Agilent 6460 series MS/MS system (Agilent Technologies, Santa Clara, CA, United States) to quantify the analytes in the ESI-positive ionization mode. The mass spectrum conditions were as follows: a capillary voltage of 4,000 V; a gas flow rate of 10 L/min; a nebulizer of 30 psi; a gas temperature of 350°C; and a Delta EMV(+) of 200 V. Five microliters of the samples was injected into an Agilent SB-C_18_ column (2.1 mm × 50 mm, 1.8 μm particles). The analytes were separated using the gradient elution method, and the mobile phase was composed of water (0.05% V/V formic acid) (A) and acetonitrile (B) at a flow rate of 0.4 mL/min (0–1 min, 10–10% B; 1–5 min, 10–90% B; 5–7 min, 90–90% B; and 7–7.1 min, B 90–10%; rebalance for 2 min). The multiple reaction-monitoring parameters are shown in Table 1.

**TABLE 1:** Multiple reaction monitoring parameters of the mass spectrometer detector.

Component	Signature peptides	Molecular weight	Parent ion	Product ion	Fragmentor (V)	Collision energy (eV)
Glycyrrhizic acid	\	822.93	823.3	453.2	210	34
Glycyrrhetinic acid	\	470.68	471.2	189.1	190	37
Dehydrotumulosic acid	\	484.71	467.2	311.1	165	26
Isoliquiritin	\	418.39	419.0	257.0	115	17
Liquiritin	\	418.39	419.0	137.0	125	24
Atractylenolide III	\	248.32	249.0	231.1	65	7
Cinnamic acid	\	148.16	149.1	103.0	70	23
Schisandrin (IS)	\	432.50	433.0	384.1	80	20
MRP2	LTIIPQDPILFSGSLR	1,770.08	885.7	1,329.9	200	25
OCT1	GVALPETIEEAENLGR	1,697.84	849.7	1,357.8	180	29
NTCP	AAATEDATPAALEK	1,358.42	680	915.5	140	18
IS	AAATEDATPAALE ***K*** ^*^	1,366.42	684	923.0	140	23
OATP1A4	TFQFPGDIESSK	1,355.45	678.6	832.3	160	19
MATE1	LLLSGFQEELR	1,304.48	652.9	965.0	140	24
BSEP	STALQLIQR	1,029.19	515.5	529.5	130	17
MDR1	NTTGALTTR	934.00	467.9	719.4	110	14
OATP1A1	EENLGITK	903.00	452.3	468.1	120	8
OATP1A2	SVQPELK	799.91	400.8	486.3	110	9
UGT1A1	TYPVPFQR	1,336.48	504.5	547.1	140	19

#### 2.4.2 Sample preparation

A plasma sample of 50 µL was placed in a 1.5 mL centrifuge tube with 10 µL of schisandrin (IS) solution (50 ng/mL) and mixed for 5 min. The samples were successively extracted with 500 µL of ethyl acetate and treated with 250 µL of methanol, which were then mixed for 5 min and centrifuged at 4°C for 10 min (at 18,000 rpm). All the supernatant was absorbed into another 1.5 mL centrifuge tube, dried under a gentle stream of nitrogen at 37°C, and then added with 50 µL of 50% methanol for reconstruction. After being centrifuged at 4°C for 10 min (at 18,000 rpm), 5 µL of the supernatant was extracted and injected into a chromatographic column for analysis, and the chromatograms were recorded.

#### 2.4.3 Method validation

The bioanalytical method was validated based on the current US FDA Bioanalytical Method Validation Guidance for Industry (Gu, 2020), with validation parameters such as specificity, linearity, precision and accuracy, recovery, matrix effect, and stability being involved.

##### 2.4.3.1 Specificity

The specificity of endogenous interference was examined by chromatograms of six batches of blank rat plasma, blank plasma with the seven components and internal standard, and plasma samples after the oral administration of LGZGD for 4 h.

##### 2.4.3.2 Linearity

The calibration curve was obtained by measuring the blank plasma samples with analytes at eight concentration levels and by analyzing the peak area ratio of the components to IS using weighted (1/x^2^) least squares linear regression. The LLOQ was defined as the lowest quantifiable calibration concentration with an acceptable accuracy within ±20%, which was hereby determined at the lowest concentration with a signal-to-noise ratio (S/N) of 10.

##### 2.4.3.3 Precision and accuracy

The precision and accuracy were evaluated using six duplicated, three (low, medium, and high) quality control samples on the same day and three batches of samples for three consecutive days, respectively. The accuracy was expressed as a relative error percentage (RE, %), while the intra-day and inter-day precisions were evaluated as a relative standard deviation percentage (RSD).

##### 2.4.3.4 Extraction recovery and matrix effect

At three quality control (QC) levels, the extraction recovery was determined by comparing the ratio of the peak area to the internal standard in the blank plasma sample with the analyte added before extraction and after extraction. The matrix effect was measured at three QC levels by comparing the peak areas of the extracted blank plasma samples with the analyte (six batches of rat plasma) and the corresponding pure standard solution.

##### 2.4.3.5 Stability

Stability refers to the ability of the components to retain their chemical properties in the matrix. To analyze the stability of all components in the plasma, six replicates of QC samples at three concentrations were hereby prepared under each condition. The stability of the QC samples at three concentrations was analyzed under different storage conditions: −80°C for 30 days, after three freeze–thaw cycles, and 24 h in an auto sampler at 4°C.

### 2.5 Application to pharmacokinetic analysis

The rats were randomly divided into the normal group and the model group. According to the preliminary experiment, the dose was adjusted to about eight times the normal dose (Gu, 2020; Li et al., 2023). The normal and NAFLD groups were each orally treated with 23 g/kg of extract (equivalent to LGZGD raw herbs 131 g/kg), and the content of each analyte was determined. The animal research program was approved by the Animal Committee of Shanghai University of Traditional Chinese Medicine. At 0, 0.083, 0.25, 0.5, 0.75, 1, 2, 4, 6, 8, 10, 12, 24, and 48 h after dosing, 150 µL blood samples were collected from the rats' orbital venous plexus into centrifuge tubes containing 10 IU of sodium heparin. After being centrifuged at 5,000 rpm (4°C) for 10 min, the plasma was transferred to 1.5 mL polypropylene tubes and stored at −80°C until the plasma samples were analyzed.

Eight weeks after modeling, the blood samples were collected in the coagulation vessels of the model group and the normal group, which were then centrifuged at 4°C for 10 min (5,000 rpm) to obtain the serum samples. Total cholesterol (TC), triglyceride (TG), high-density lipoprotein cholesterol (HDL-C), and low-density lipoprotein cholesterol (LDL-C) were determined to evaluate the modeling efficiency. Besides, the liver samples were isolated and stored in 4% paraformaldehyde for histological examination. Serum TG, TC, HDL-C, and LDL-C were measured by using a chemical analyzer (Hitachi 7080, Japan). The liver tissue blocks fixed with 4% of formaldehyde solution were embedded in paraffin, sectioned, stained with hematoxylin and eosin (H&E), and observed under an optical microscope.

### 2.6 Determination of UGT1A1 and nine other transporter proteins in the liver

Herein, a comprehensive study was conducted to detect the expressions of UGT1A1 and nine other transporters in the liver of the NAFLD model group and normal rats, which were successfully determined. Previous methods of protein quantification were immunological, which however require antibodies that are frequently unavailable, and even when available, are often of uncertain specificity. Additionally, the UPLC-MS/MS method was hereby used to detect the expression level of metabolic enzymes, which is provided with advantages in absolute accuracy, stability, operability, and efficiency when compared with the traditional quantitative methods (Yi et al., 2018).

#### 2.6.1 Instrumentation and chromatographic conditions

The Agilent 1290 series UPLC system and Agilent 6460 series MS/MS system (Agilent Technologies, Santa Clara, CA, United States) were hereby combined to quantify the signature peptides in the ESI-positive ionization mode. The conditions of mass spectrometry were as follows: a capillary voltage of 2,000 V; a gas flow of 8 L/min; a nebulizer of 30 psi; a gas temperature at 300°C; and a Delta EMV(+) of 400 V. Five milliliters of the sample was injected into the column (Agilent SB-C_18_ column, 2.1 mm × 50 mm, 1.8 µm particles) and eluted at 0.4 mL/min with a gradient elution of water (with 0.05% v/v formic acid) (A) and acetonitrile (B) (0–1 min, 5–5% B; 1–4 min, 5–60% B; 4–5 min, 60–5% B; and re-equilibration for 3 min). According to previous experiments by the research group of Yi et al. (2018), the sequences of characteristic peptides corresponding to UGT1A1 and the nine other transporters are listed in Table 1.

#### 2.6.2 Sample preparation

Total membrane proteins were isolated from the liver tissue using the Native Membrane Protein Extraction Kit protocols (three replicates), and the protein concentration was detected by the BCA Protein Assay Kit. The preparation process was based on the previous experiments by the present researchers (Yi et al., 2018; Ma et al., 2021). In simple terms, 10 µL of 5 mg/mL liver cell proteins was cultured with 20 µL of dithiothreitol (100 mM) and 50 µL of ammonium bicarbonate buffer (50 mM, pH = 7.8). After being incubated at 95°C for 5 min, 20 µL of iodoacetamide (20 mM) was added and cultured at 37°C for 20 min away from light. The sample was concentrated, and iced methanol (0.5 mL), chloroform (0.2 mL), and water (0.2 mL) were added, which was then centrifuged at 16,000 *g* for 5 min at 4°C. Afterward, the supernatant was removed, and the sample was washed once with 0.25 mL of cold methanol and recombined with 40 µL of recombination solution (equal volume 3% sodium deoxycholate (w/v) and 5 mM ammonium bicarbonate buffer). Finally, the protein samples were digested with 10 µL of trypsin. The ratio of protein to trypsin was 25:1 (w/w). After incubation at 37°C for 24 h, the digestion reaction was terminated by 60 µL of IS cocktail. The samples were centrifuged at 18,000 *g* for 10 min at 4°C, and 5 µL of the sample was injected in the UPLC-MS/MS system.

### 2.7 Data processing and statistical analysis

Important pharmacokinetic parameters, which included the maximum plasma concentration (*C*_max_), time to maximum concentration (*T*_max_), half-life (*t*_1/2_), area under the curve (AUC_0-t_), and total area under the curve (AUC_0-∞_), were hereby calculated using the DAS 3.2.8 software. Meanwhile, the pharmacokinetic data of the normal and model rats were compared using the SPSS software (version 21.0). Besides, Spearman rank bivariate correlation coefficient was used to determine the correlation between AUC values and UGT1A1 and nine other transporters. All the calculated data were expressed as mean ± SD. When *p* < 0.05, the difference was considered significant and statistically significant; and when *p* < 0.01 or <0.001, the difference was considered very significant.

## 3 Results

### 3.1 Serum biochemistry and histopathological examination

**FIGURE 2:**
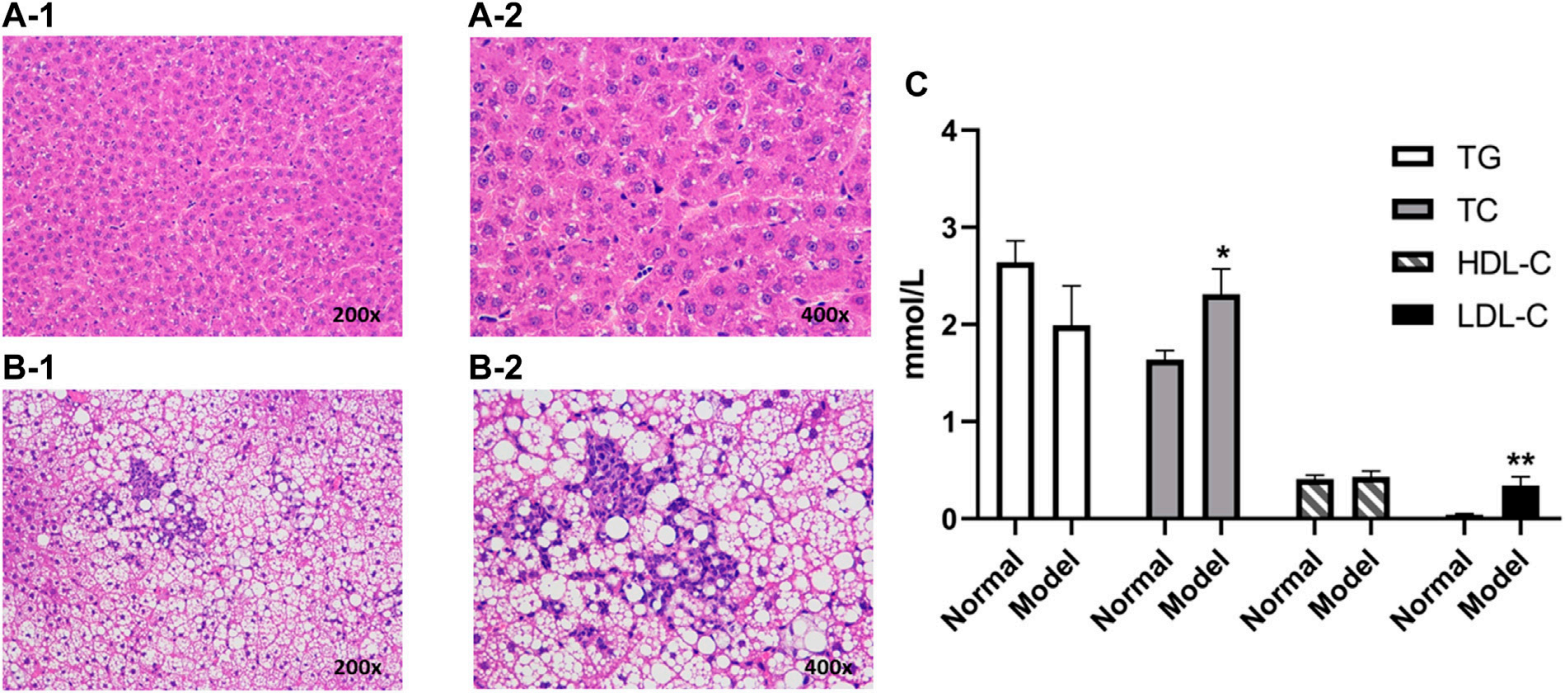
Liver histopathology of rats with and without NAFLD. Representative histopathology of liver sections from control (non-NAFLD) rats: **(A-1)** ×200 magnification and **(A-2)** ×400 magnification, and from NAFLD rats: **(B-1)** ×200 magnification and **(B-2)** ×400 magnification. The serum biochemical indexes of the model group were compared with those from the normal group **(C)**. Liver sections were stained with hematoxylin and eosin prior to microscopy (**p < 0.01 and *p < 0.05, which compared with the control group).

The weights of the model group rats were significantly increased after being continuously fed a high-fat diet for 8 weeks. The serum biochemical indexes of the NAFLD group were compared with those of the normal group (Figure 2C), and the TC of the model group (*p* < 0.05) and the LDL-C (*p* < 0.01) were significantly increased, but no significant difference was observed between TG and HDL-C. Besides, liver pathological sections were observed under the microscope (Figures 2A, B). The liver cells in the normal group were arranged neatly, with no obvious lesions, were polygonal and with well-defined boundaries, having nuclei located in the center of the cells, with homogeneous and rich cytoplasm, and no fat droplet deposition in the liver cells. In the NAFLD group, the hepatocytes were disordered and the volume of the hepatocytes was significantly enlarged and swollen, which showed diffused steatosis. Lipid vacuoles of different sizes and numbers were observed in the hepatocytes, and the nuclei were displaced and partially dissolved. Similar to the biochemical and pathological aspects of simple NAFLD (Anstee and Goldin, 2006), and in line with the characteristics of NAFLD, the model was successfully prepared.

### 3.2 Pharmacokinetic study of LGZGD in NAFLD and normal rat models

#### 3.2.1 Method validation

##### 3.2.1.1 Specificity

**FIGURE 3:**
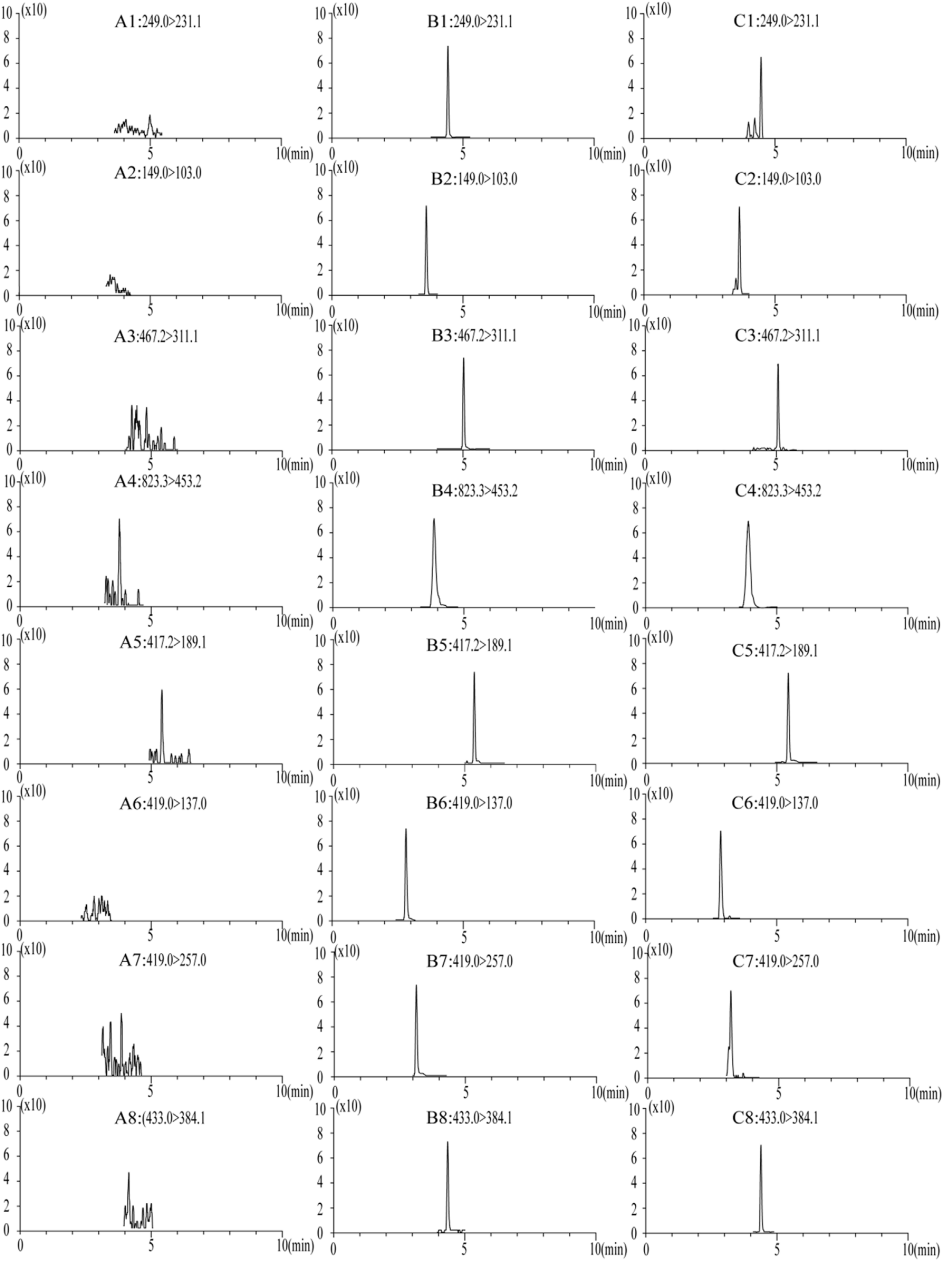
MRM chromatograms of blank plasma samples: **(A)** blank spiked with combined standard solutions of the seven analytes and IS, **(B)** rat plasma samples at 4 h after oral administration of LGZGD **(C)**. 1, atractylenolide III; 2, cinnamic acid; 3, dehydrotumulosic acid; 4, glycyrrhizic acid; 5, glycyrrhetinic acid; 6, liquiritin; 7, isoliquiritin; and 8, schisandrin (IS).

No endogenous interference was found in the retention time of the analyte and internal standard in the blank plasma of the six different rats, which proved the specificity of this method. Figure 3 presents the typical chromatograms of blank rat plasma, blank samples supplemented with seven compounds and internal standards, and rat plasma samples after the oral administration of LGZGD for 4 h.

##### 3.2.1.2 Linearity

The relationship curve between the peak area ratio (*y*-axis) of each analyte and IS and the corresponding nominal concentration (*x*-axis) of the analyte was plotted by weighted (1/x^2^) least square linear regression. All standard curves showed a good linearity with the correlation coefficient (*r* > 0.9952). The calibration curves, linear ranges, LLOQ, and correlation coefficients of the seven components are shown in Table 2, where the stablished method can be seen to meet the requirements of the quantitative determination of LGZGD in pharmacokinetic studies.

**TABLE 2:** Regression equations, linear range, and LLOQ of the seven components in rat plasma.

Component	Regression equation	Correlation coefficient(r)	Linear range (ng/mL)	LLOQ (ng/mL)
Glycyrrhizic acid	y = 0.0009x + 0.0212	0.9957	2.66–5440	1.97
Glycyrrhetinic acid	y = 0.0137x + 0.1468	0.9952	2.00–8180	1.75
Dehydrotumulosic acid	y = 0.0094x + 0.1236	0.9966	1.23–5050	0.32
Isoliquiritin	y = 0.126x + 0.7072	0.9964	1.18–4850	0.14
Liquiritin	y = 0.0047x + 0.0991	0.9973	4.83–9900	3.99
Atractylenolide III	y = 0.0707x + 0.8165	0.9952	2.63–5390	1.80
Cinnamic acid	y = 0.0321x + 0.4800	0.9982	2.65–5430	0.99

##### 3.2.1.3 Precision and accuracy

The precision and accuracy are summarized in Table 3. The intra-day and inter-day precision (relative standard deviation) was less than 14.9% and the accuracy (relative error) was less than 12.8%. All results were within acceptable standards according to the guidelines for bioassay methods.

**TABLE 3:** Summary of accuracy, precision, recovery, and matrix effect of the seven components in rat plasma (n = 6).

Component	Concentration (ng/mL)	Precision and accuracy				Recovery (%)		Matrix effect (%)	
		Intra‐day (%)		Inter‐day (%)					
		RSD	RE	RSD	RE	Mean	RSD	Mean	RSD
Glycyrrhizic acid	5.31	7.3	−0.1	3.7	−6.6	97.8	4.8	107.2	5.7
	170.0	9.1	−6.1	5.1	−12.8	72.0	2.5	111.2	4.1
	2,720	8.4	−7.6	8.6	2.0	79.6	2.3	112.6	2.4
Glycyrrhetinic acid	3.99	13.4	7.4	13.1	−9.9	67.3	9.7	110.5	3.9
	127.8	8.0	4.4	6.8	−6.3	70.2	7.7	89.9	1.8
	2,045	5.3	−10.2	7.3	2.0	71.1	6.3	91.0	2.5
Dehydrotumulosic acid	4.93	3.9	11.0	10.3	−3.4	71.3	12.1	96.1	4.8
	157.8	6.3	1.5	2.9	−1.7	63.8	9.1	83.4	2.4
	2,525	5.0	3.7	13.0	5.5	67.1	5.6	87.9	12.6
Isoliquiritin	4.74	14.2	−4.0	2.0	−3.0	91.9	7.9	104.2	12.5
	151.6	7.0	−3.0	10.2	3.1	92.0	6.3	100.7	1.8
	2,425	5.3	−11.1	14.9	3.9	90.1	3.1	106.8	1.4
Liquiritin	4.83	11.2	8.5	14.1	−9.0	86.4	2.9	110.3	3.2
	154.7	7.3	−1.8	14.2	−4.6	86.9	6.4	107.2	1.9
	2,475	6.1	0.2	2.4	−0.4	87.2	2.4	105.9	5.4
Atractylenolide III	5.26	12.8	5.7	13.5	−9.4	80.0	2.0	109.2	4.1
	168.4	6.5	4.5	11.8	−10.9	87.1	6.4	102.0	2.4
	2,695	3.8	−3.8	9.8	−0.8	92.0	5.7	101.6	1.2
Cinnamic acid	5.30	9.4	−0.4	6.1	10.6	70.9	3.7	105.4	3.1
	169.7	8.0	−1.3	5.2	8.8	72.2	9.0	104.9	1.8
	2,715	7.5	−1.5	6.2	−0.2	83.8	5.8	112.4	1.5

##### 3.2.1.4 Extraction recovery and matrix effect

Table 3 summarizes the average extraction recoveries and matrix effects of seven components at three QC levels. The recoveries of the three QC samples of the seven analytes range from 63.8 to 92.0%, while the matrix effect range from 83.4 to 112.6%. These results indicate the acceptable recovery and matrix effect of the liquid–liquid extraction combined with the protein precipitation method in the present study.

##### 3.2.1.5 Stability

The data in Table 4 show that the seven components in the rat plasma are stable in the plasma samples maintained at −80°C for 30 days, during the three freeze–thaw cycles, and in the 24 h in an auto sampler at 4°C—stable and consistent with the analytical criteria during the whole experiment.

**TABLE 4:** Stability of the seven components in rat plasma at three quality control levels (n = 6).

Component	Concentration (ng/mL)	Stability during 24 h at 4°C		Three freeze–thaw cycles		−80°C for 30 days	
		RSD (%)	RE (%)	RSD (%)	RE (%)	RSD (%)	RE (%)
Glycyrrhizic acid	5.31	11.0	−1.6	13.3	−5.9	3.6	−1.0
	170.0	14.6	−5.6	12.6	−3.7	7.1	1.7
	2,720	13.7	−3.3	14.2	9.7	2.7	2.7
Glycyrrhetinic acid	3.99	13.4	5.2	11.4	−0.4	3.6	−5.2
	127.8	2.5	6.7	7.9	−7.7	2.4	−4.9
	2,045	8.7	−8.9	5.6	2.2	5.9	2.6
Dehydrotumulosic acid	4.93	5.0	3.8	14.6	−12.8	6.4	3.2
	157.8	3.6	1.6	2.8	−4.3	8.2	10.2
	2,525	8.1	−2.3	7.6	5.5	3.9	3.0
Isoliquiritin	4.74	5.9	1.3	11.3	−6.8	11.7	−1.1
	151.6	5.6	11.0	8.6	−3.0	7.3	−2.1
	2,425	7.8	−12.7	6.7	3.9	5.5	4.0
Liquiritin	4.83	8.7	1.4	7.2	10.5	5.5	−4.1
	154.7	5.4	5.4	10.6	−1.8	7.4	3.1
	2,475	8.7	−3.0	7.9	4.1	5.6	−1.2
Atractylenolide III	5.26	14.6	6.4	10.4	13.3	10.6	−4.4
	168.4	6.0	5.2	7.4	−9.5	4.4	−3.5
	2,695	7.8	−4.0	8.9	−3.8	7.2	14.1
Cinnamic acid	5.30	14.7	8.4	11.8	−0.4	2.4	−8.0
	169.7	5.4	13.9	4.5	0.7	6.6	3.6
	2,715	14.7	−3.7	7.6	−11.1	5.7	−2.6

### 3.3 Pharmacokinetic study of LGZGD in normal and NAFLD rat models

**FIGURE 4:**
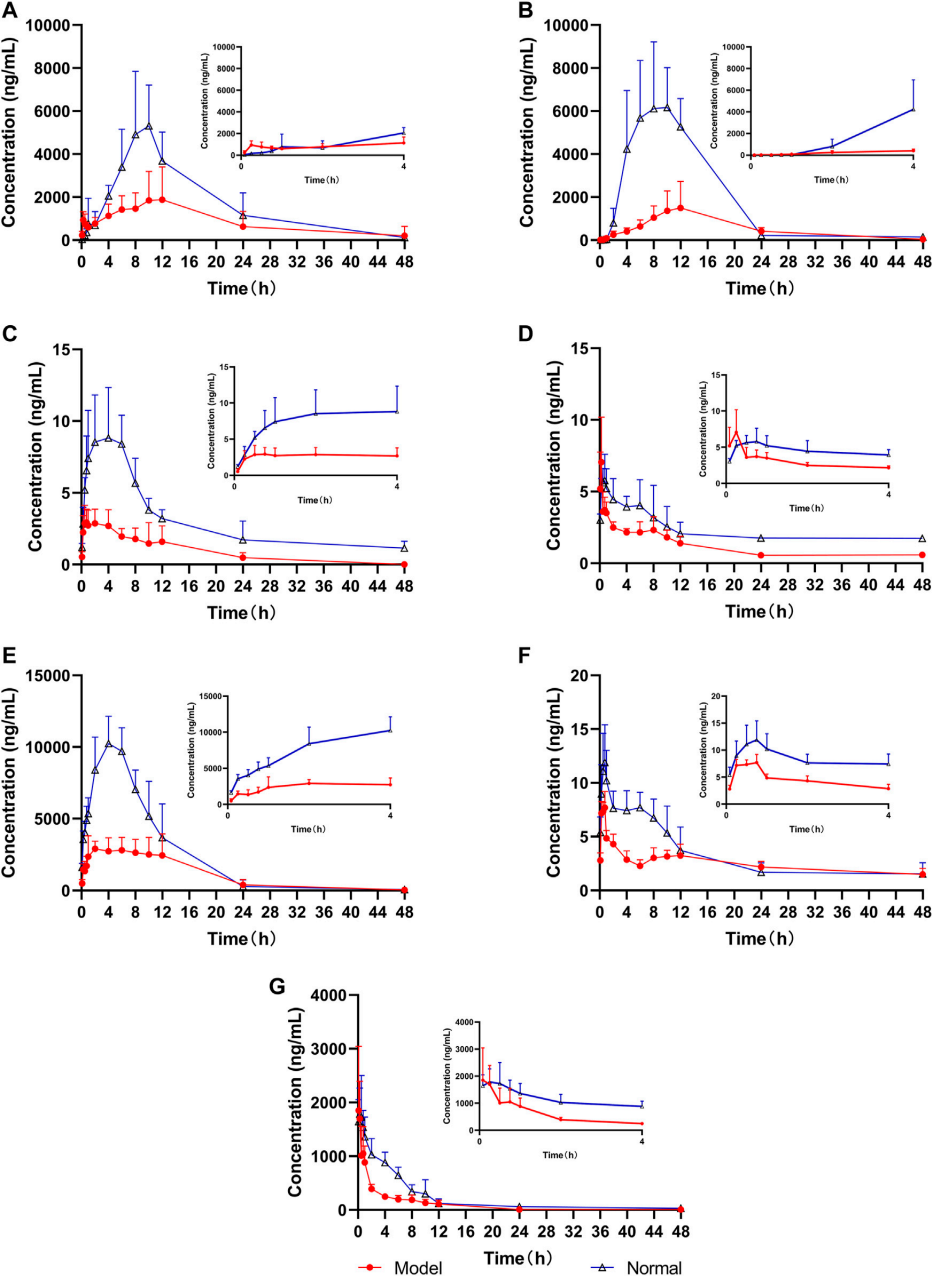
Mean concentration–time curves of the seven analytes in rat plasma (n = 6), **(A)** glycyrrhizic acid, **(B)** glycyrrhetinic acid, **(C)** dehydrotumulosic acid, **(D)** isoliquiritin, **(E)** liquiritin, **(F)** atractylenolide III, and **(G)** cinnamic acid.

The UPLC-MS/MS method was successfully used to compare the pharmacokinetics of the seven components of LGZGD in the normal and NAFLD rats. The glycyrrhizic acid, glycyrrhetinic acid, dehydrotumulosic acid, isoliquiritin, liquiritin, atractylenolide III, and cinnamic acid were detected in the plasma of normal and NAFLD rats. The mean plasma concentration–time curve is shown in Figure 4, and the corresponding pharmacokinetic data are listed in Table 5. The pharmacokinetic behavior of the LGZGD components in the normal group was significantly different from that in NAFLD rats.

**TABLE 5:** Pharmacokinetic parameters of the seven components in male SD rats after the oral administration of LGZGD (mean ± SD, n = 6).

Component	Groups	*t*_1/2_(h)	*T*_max_(h)	*C*_max_ (ug/L)	AUC_0-t_ (ug/L*h)	AUC_0-∞_(ug/L*h)	CLz/F (L/h/kg)	MRT_0-t_(h)
Glycyrrhizic acid	Normal	6.3 ± 3.0	10.4 ± 1.7	6415.5 ± 2238.9	81042.3 ± 10475.2	82667.3 ± 10040.9	281.8 ± 36.8	13.7 ± 3.3
	Model	5.4 ± 1.4	8.1 ± 4.9	2243.1 ± 1202.0**	40701.8 ± 25060.3*	40806.7 ± 25026.0**	701.2 ± 305.3*	16.0 ± 8.0
Glycyrrhetinic acid	Normal	6.0 ± 2.1	9.3 ± 1.6	7281.6 ± 2079.1	88249.6 ± 27217.9	89638.0 ± 28611.9	273.6 ± 64.8	10.8 ± 1.1
	Model	8.9 ± 4.5	13.2 ± 6.3	1648.9 ± 1173.3***	25626.1 ± 14778.6**	26152.2 ± 14423.4**	1184.3 ± 716.4*	15.6 ± 2.3**
Dehydrotumulosic acid	Normal	19.0 ± 12.2	4.0 ± 1.8	10.1 ± 3.0	141.6 ± 15.8	174.6 ± 37.2	136156.3 ± 25915.9	14.4 ± 2.7
	Model	5.0 ± 1.8*	1.8 ± 1.6	3.8 ± 0.6**	43.4 ± 12.0***	43.5 ± 12.0***	559085.2 ± 149625.6***	10.4 ± 2.2*
Isoliquiritin	Normal	9.2 ± 4.4	0.6 ± 0.1	6.1 ± 1.8	107.6 ± 21.2	166.5 ± 10.5	82306.9 ± 61479.7	19.8 ± 1.7
	Model	13.1 ± 7.5	0.7 ± 0.2	4.2 ± 0.8*	51.9 ± 7.8***	56.9 ± 10.8***	403580.1 ± 75381.0***	15.5 ± 1.8**
Liquiritin	Normal	5.2 ± 2.5	4.8 ± 1.1	10332.4 ± 1848.3	114704.1 ± 32858.5	115445.5 ± 32941.3	211.0 ± 56.8	7.7 ± 1.6
	Model	6.0 ± 3.4	6.8 ± 4.1	3741.7 ± 991.4***	52772.6 ± 22330.1**	53400.2 ± 22110.2**	491.1 ± 204.8*	10.1 ± 1.3*
Atractylenolide III	Normal	10.2 ± 4.4	0.7 ± 0.1	12.2 ± 3.4	352.5 ± 21.5	568.1 ± 234.7	149538.5 ± 21123.9	14.7 ± 1.5
	Model	18.3 ± 1.3*	0.6 ± 0.1	7.9 ± 1.3*	116.7 ± 16.1***	165.8 ± 45.1**	171606.0 ± 32478.4	19.3 ± 2.9*
Cinnamic acid	Normal	12.8 ± 3.6	0.6 ± 0.1	1930.9 ± 483.9	10094.3 ± 1770.0	10505.6 ± 1902.0	2180.2 ± 440.9	8.2 ± 1.3
	Model	6.6 ± 3.5*	0.6 ± 0.1	1190.4 ± 352.4*	4332.2 ± 1334.7***	4406.4 ± 1420.0***	5187.3 ± 1815.1**	6.2 ± 1.8

*ANOVA* test was used to calculate the significance of the differences, ***p < 0.001, **p < 0.01, and *p < 0.05, which compared with the normal group. The CLz/F is apparent clearance.

By comparing the pharmacokinetic parameters of LGZGD orally taken by NAFLD and normal rats, it was found that isoliquiritin. atractylenolide III, and cinnamic acid were rapidly absorbed in both normal and NAFLD rats, and the *T*_max_ was not significantly different between the model group and normal rats. The mean AUC_0-t_ of the model group was significantly lower than that of the normal group. Compared with the normal group, the MRT_0-t_ of glycyrrhetinic acid, atractylenolide III, and liquiritin was prolonged by 44.4, 31.3, and 31.2%, respectively, while the MRT of dehydrotumulosic acid and isoliquiritin decreased by 27.8 and 21.7%, respectively. The *C*_max_ value, AUC_0-t_, and AUC_0-∞_ of the NAFLD rats were significantly lower than those of the normal rats. When compared with that in the normal rats, the *C*_max_ value of glycyrrhizic acid, glycyrrhetinic acid, dehydrotumulosic acid, isoliquiritin, liquiritin, atractylenolide III, and cinnamic acid in NAFLD rats was decreased by 77.4, 65.0, 62.4, 31.1, 63.8, 35.2, and 38.3%, respectively, and the AUC_0*-∞*_ value decreased by 70.8, 50.6, 75.1, 65.8, 53.7, 70.8, and 58.1%, respectively.

The blood concentration of liquiritin in LGZGD was the highest, followed by that of glycyrrhizic acid, glycyrrhetinic acid, cinnamic acid, atractylenolide III, dehydrotumulosic acid, and isoliquiritin. In the NAFLD rats, the clearance rate of glycyrrhizic acid, glycyrrhetinic acid, dehydrotumulosic acid, isoliquiritin, liquiritin, atractylenolide III, and cinnamic acid was significantly higher than that of the normal group. There was also a bimodal phenomenon between glycyrrhetinic acid and isoliquiritin (Xu et al., 2018; Shen et al., 2019), which might have been caused by gastric emptying separation, enterohepatic circulation, and reabsorption (Deng et al., 2008; Zhu et al., 2010). Further research should be carried out to determine the specific mechanism. All these results suggest lower systemic exposure of LGZGD in NAFLD rats, which might provide useful evidence for the pathological changes in NAFLD rats and the clinical application of LGZGD.

### 3.4 Expression of UGT1A1 and nine other transporter proteins in NAFLD and normal rats’ liver

**FIGURE 5:**
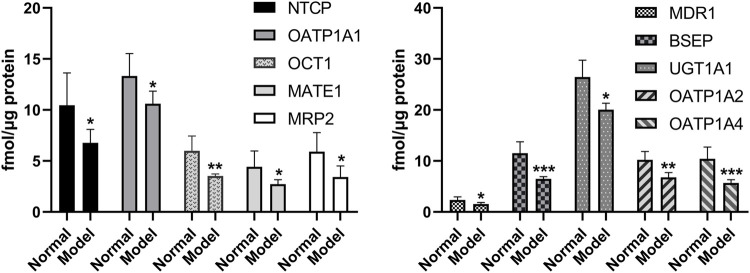
Expression levels of metabolic enzyme UGT1A1 and nine transporters in the liver of model group and normal group (*p < 0.05, **p < 0.01, and ***p < 0.001, which compared with the normal group).

The quantitative results of UGT1A1 and the nine transporters are shown in Figure 5. The expression levels of UGT1A1 and NTCP, OATP1A1, OCT1, MATE1, MRP2, MDR1, BSEP, OATP1A2, and OATP1A4 of the nine transporters in the liver of the model group are significantly lower than those of the normal group, which is consistent with the reports of previous studies (Kim et al., 2004; Kirpich et al., 2011). Uridine diphosphate glucuronosyltransferase (UGT) is a common phase II metabolic enzyme that plays an important role in drug binding reactions. Meanwhile, OCT1, NTCP, OATP1A1, OATP1A2, and OATP1A4 are uptake transporters, whereas MRP2, BSEP, MATE1, and MDR1 are efflux transporters (Chen L. et al., 2019). The expressions of UGT1A1 metabolic enzyme and nine transporters are decreased in NAFLD rats, indicating that the metabolism of the drug components absorbed into the blood was inhibited in the model group, and the drug uptake and excretion were also inhibited. When compared with the normal group, UGT1A1, NTCP, OCT1, MATE1, MDR1, BSEP, and OATP1A2 in the NAFLD group are significantly decreased by 24.1, 35.3, 30.8, 38.6, 34.3, 43.7, and 33.8% (*p* < 0.05), respectively.

### 3.5 Correlation between AUC values and expression levels of metabolic enzyme and transporters

The AUC is an important indicator of drug absorption. In this study, a significant decrease in UGT1A1 and the nine other transporters was observed in the NAFLD rats. In order to further understand the reasons for the pharmacokinetic differences, a correlation analysis between the expression levels of the active ingredients and UGT1A1 and transporters in the model group and the normal group was conducted, and the results are shown in Table 6 and Supplementary Figures S1–S50.

**TABLE 6:** Correlation results of AUC values with metabolic enzymes and transporters (**p < 0.01 and *p < 0.05).

Component	Correlation coefficient ρ									
	UGT1A1	NTCP	OATP1A1	OCT1	MATE1	MRP2	MDR1	BSEP	OATP1A2	OATP1A4
Glycyrrhizic acid	0.515	0.455	0.600	0.624	0.491	0.612	0.681*	0.709*	0.552	0.539
Glycyrrhetinic acid	0.576	0.721*	0.867**	0.830**	0.745*	0.903**	0.772**	0.976**	0.733*	0.855**
Dehydrotumulosic acid	0.564	0.685*	0.733*	0.758*	0.648*	0.794**	0.657*	0.903**	0.697*	0.794**
Isoliquiritin	0.770**	0.661*	0.758*	0.782**	0.770**	0.673*	0.845**	0.733*	0.600	0.612
Liquiritin	0.588	0.685*	0.685*	0.527	0.539	0.527	0.547	0.745*	0.564	0.564
Atractylenolide III	0.697*	0.770**	0.867**	0.733*	0.867**	0.733*	0.802**	0.855**	0.612	0.685*
Cinnamic acid	0.733*	0.818**	0.939**	0.661*	0.721*	0.709*	0.748*	0.891**	0.733*	0.673*

The expression levels of BSEP (ρ = 0.709, ρ = 0.976, ρ = 0.903, ρ = 0.733, ρ = 0.745, ρ = 0.855, and ρ = 0.891) (*p* < 0.05) were significantly correlated with the exposure amount of the seven components *in vivo*, respectively, while those of NTCP (ρ = 0.721, ρ = 0.685, ρ = 0.661, ρ = 0.685, ρ = 0.770, and ρ = 0.818) (*p* < 0.05) and OATP1A1 (ρ = 0.867, ρ = 0.733, ρ = 0.758, ρ = 0.685, ρ = 0.867, and ρ = 0.939) (*p* < 0.05) were significantly correlated with the exposure amount of glycyrrhetinic acid, dehydrotumulosic acid, isoliquiritin, liquiritin, atractylenolide III, and cinnamic acid *in vivo*, respectively, and those of MDR1 were significantly correlated with the AUC values of glycyrrhizic acid (ρ = 0.681), glycyrrhetinic acid (ρ = 0.772), dehydrotumulosic acid (ρ = 0.657), isoliquiritin (ρ = 0.845), atractylenolide III (ρ = 0.802) (*p* < 0.01), and cinnamic acid (ρ = 0.748) (*p* < 0.05). In addition, the expression levels of NTCP and OATP1A1 were significantly correlated with the AUC values of glycyrrhetinic acid (ρ = 0.721 and ρ = 0.867), dehydrotumulosic acid (ρ = 0.685 and ρ = 0.733), isoliquiritin (ρ = 0.661 and ρ = 0.758), liquiritin (ρ = 0.685 and ρ = 0.685), atractylenolide III (ρ = 0.770 and ρ = 0.867) (*p* < 0.01), and cinnamic acid (ρ = 0.818 and ρ = 0.939) (*p* < 0.01). The expression levels of OCT1, MATE1, and MRP2 were found related to the AUC values of glycyrrhetinic acid (ρ = 0.830, ρ = 0.745, and ρ = 0.903), dehydrotumulosic acid (ρ = 0.758, ρ = 0.648, and ρ = −0.794), isoliquiritin (ρ = 0.782, ρ = 0.770, and ρ = 0.673), atractylenolide III (ρ = 0.733, ρ = 0.867, and ρ = 0.733), and cinnamic acid (ρ = 0.661, ρ = 0.721, and ρ = 0.709) (*p* < 0.05).

Besides, the expression levels of OATP1A4 were significantly correlated with the AUC values of glycyrrhetinic acid (ρ = 0.855) (*p* < 0.01), dehydrotumulosic acid (ρ = 0.794) (*p* < 0.01), atractylenolide III (ρ = 0.685) (*p* < 0.05), and cinnamic acid (ρ = 0.673) (*p* < 0.05), while those of OATP1A2 were significantly correlated with the AUC values of glycyrrhetinic acid (ρ = 0.733) (*p* < 0.05), dehydrotumulosic acid (ρ = 0.697) (*p* < 0.05), and cinnamic acid (ρ = 0.733) (*p* < 0.05).

## 4 Discussion

As mentioned previously, NAFLD is a common chronic liver disease (Parlati et al., 2021) and the lack of safe and effective drugs for its thorough treatment has made related cardiovascular diseases a major problem of chronic diseases (Lou et al., 2020; Gu et al., 2021). Herein, the animal models were divided into the genetic model, diet model, and combination model of genetic and diet factors (Takahashi et al., 2012). High-fat diets were found more closely related to the pathogenesis of human patients, and this method was more widely used in the manufacture of animal models of NAFLD. The NAFLD model of rats was established by high-fat diets. Under the microscope, the liver cells in the model group appeared disordered, the volume of liver cells had increased significantly, lipid vacuoles of different sizes were observed in the cells, and the nucleus was displaced and partially dissolved. In terms of biochemistry and pathology, the TC and HDL-C of the model rats were significantly different from those of the normal rats, similar to those of NAFLD alone (Anstee and Goldin, 2006), in line with the characteristics of NAFLD. Thus, the model was successfully prepared and used in pharmacokinetic research.

Liquid–liquid extraction can effectively reduce the endogenous effect. In the early stage of the experiment, organic reagents were used to precipitate protein, but the recoveries of dehydrotumulosic acid and glycyrrhizic acid were not higher than 50%. Methanol, ethyl acetate, and methanol combined with ethyl acetate were subsequently investigated, and the recovery of glycyrrhizic acid and dehydrotumulosic acid was found to be the highest when the samples were extracted with 500 µL ethyl acetate and treated with 250 µL methanol successively. The pharmacokinetics of seven LGZGD components in the normal and NAFLD rats were successfully compared by using the UPLC-MS/MS method. The pharmacokinetic behavior of the seven components was significantly different between the normal group and the fatty liver model rats. The AUC_0-∞_ and *C*_max_ of glycyrrhizic acid, glycyrrhetinic acid, dehydrotumulosic acid, isoliquiritin, liquiritin, atractylenolide III, and cinnamic acid in the plasma of NAFLD rats were significantly lower than those of normal rats. This is basically the same trend in the normal group and the NAFLD rats. The mean residence time of atractylenolide III, glycyrrhizic acid, glycyrrhetinic acid, and liquiritin was increased in the NAFLD group when compared with the normal group. The mean retention time of isoliquiritin and dehydrotumulosic acid was decreased. In the rat model of NAFLD, the scavenging rates of seven analytes were increased when compared with the normal group, while the *in vivo* absorption of atractylenolide III, dehydrotumulosic acid, and glycyrrhetinic acid was enhanced. The absorption of glycyrrhizic acid, liquiritin, and isoliquiritin was inhibited when compared with that in the normal group. The pharmacokinetics of the main bioactive components of *Ilex hainanensis* extract after oral administration to normal and NAFLD rats were also studied (Yang et al., 2013). The AUC and *C*_max_ of chlorogenic acid, kaempferol 7-O-β-D-glucoside, and ilexgenin A was decreased significantly under the plasma concentration–time curve. Kaempferol 7-O-β-D-glucoside and ilexgenin A increased the MRT in NAFLD rats. This pharmacokinetic feature is similar to the hereby observed phenomenon.

Changes of enzymes and transporters in the rat liver directly affect drug absorption and metabolism (Fisher et al., 2009; Li et al., 2011; Müller and Fromm, 2011). Studies have shown that protein transporters such as MDR1, NTCP, OATP1A2, OCT1, and MATE1 matter considerably in the metabolism of the components in the liver (Niemela et al., 2000; Nies et al., 2011; Koepsell, 2013; König et al., 2013). Most components of LGZGD are similar to the metabolic process of bilirubin (Wang et al., 2015; Yi et al., 2018), and its structure contains hydroxyl groups, where UGT1A1-mediated glucuronic acid coupling reaction can occur. The results of UGT1A1 and the nine other transporters in the NAFLD rats showed that the metabolism of drug components absorbed into the blood, and drug uptake and excretion were inhibited in the model group. The OCT1, NTCP, OATP1A1, OATP1A2, and OATP1A4 promoted the drug uptake transporter (Kim et al., 2004), the NAFLD model was reduced, and the drug absorption was restrained. The exposure characteristics of LGZGD active components were correlated with the expression levels of UGT1A1 and the nine transporters, with glycyrrhetinic acid, dehydrotumulosic acid, isoliquiritin, atractylenolide III, and cinnamic acid being correlated the most. The AUC values of glycyrrhetinic acid, dehydrotumulosic acid, isoliquiritin, atractylenolide III, and cinnamic acid were compared with those of NTCP, OCT1, MATE1, MRP2, MDR1, and BSEP, which were found positively correlated, indicating an increase in the excretion levels of glycyrrhetinic acid, dehydrotumulosic acid, isoliquiritin, atractylenolide III, and cinnamic acid when the expression levels of NTCP, OCT1, MATE1, MRP2, MDR1, and BSEP were inhibited. The contents of glycyrrhetinic acid, dehydrotumulosic acid, isoliquiritin, atractylenolide III, and cinnamic acid in the blood were decreased. The metabolism of LGZGD active components in the NAFLD rats require further studies.

It has also been shown that hepatic cytochrome P450 expression is significantly higher in NAFLD rats than in normal rats, which led to faster metabolism of the drug in the liver of NAFLD rats, (Prompila et al., 2008; Li et al., 2011), consistent with the pharmacokinetic behavior we observed in the model group of rats. Besides, it has been confirmed by literature reports (Lickteig et al., 2007; Kolodziejczyk et al., 2019) that the upregulated expressions of hepatic efflux transporters MRP3, MRP4, and BCRP in NAFLD rats might accelerate the excretion of drugs into the blood of NAFLD rats, resulting in reduced drug exposure in model group rats, which is consistent with the observed enhancement of the elimination rate in the model group. Further exploration should be carried out on whether the pharmacokinetic difference between the NAFLD group and the normal group after oral administration of LGZGD is related to the levels of liver phase II metabolic enzymes and transporters. The composition and function of gut microbiota are influenced by a variety of host and environmental factors, which include the diet. Additionally, the study showed that NAFLD rats had different microbiome compositions when compared with those fed on a controlled diet, and changes in the intestinal microbiome were related to metabolic parameters. The changes in intestinal flora in the model group might affect drug absorption of rats, resulting in significantly lower AUC in the NAFLD group than in the normal group. These differences might be attributed to the changes in the absorption mechanism of these components caused by the altered pathological status. The expression levels of metabolic enzymes UGT1A1 and the nine transporter proteins, that is, NTCP, OATP1A1, OCT1, MATE1, MRP2, MDR1, BSEP, OATP1A2, and OATP1A4, were significantly lower in NAFLD rats than in normal rats. The results suggest the potential application of UGT1A1 and the nine transporter proteins as biological markers for the evaluation of the ameliorative effect of LGZGD on NAFLD. However, the mechanism of the decreased absorption of the seven analytes after the oral administration of LGZGD in NAFLD rats require further studying.

## 5 Conclusion

An efficient and stable UPLC-MS/MS method was used to simultaneously determine seven components in rat plasma, which were successfully applied to study the pharmacokinetics of LGZGD in normal rats and NAFLD rats. It is also the first time to compare the pharmacokinetic characteristics of the seven components between normal and NAFLD rats after LGZGD administration. After LGZGD administration, the pharmacokinetic profiles of the seven compounds were significantly altered in NAFLD rats when compared to normal rats, which might be attributed to the decreased expression of UGT1A1 metabolizing enzymes and the nine transporters in NAFLD rats. The results indicate the applicability of UGT1A1 and the nine other transporters as potential biological markers for evaluating the ameliorative effects of LGZGD in NAFLD. Additionally, it is suggested that in hepatic pathological states, attention should be paid to the effects of abnormal drug metabolism on hepatic drug enzymes to avoid hepatotoxicity. Overall, the hereby obtained information can provide a meaningful basis for the clinical development of a dosing regimen for the treatment of NAFLD.

## Data Availability

The original contributions presented in the study are included in the article/Supplementary Material. Further inquiries can be directed to the corresponding authors.
