# Supplementary material for: Comparison of pharmacokinetic profiles of seven major bioactive components in normal and non-alcoholic fatty liver disease (NAFLD) rats after oral administration of Ling-Gui-Zhu-Gan decoction by UPLC-MS/MS
Source: Front Pharmacol. 2023 May 4;14:1174742. doi: 10.3389/fphar.2023.1174742 (PMC10192568; doi:10.3389/fphar.2023.1174742)
Supplement: Supplementary file 1 [file DataSheet1.DOCX]

**
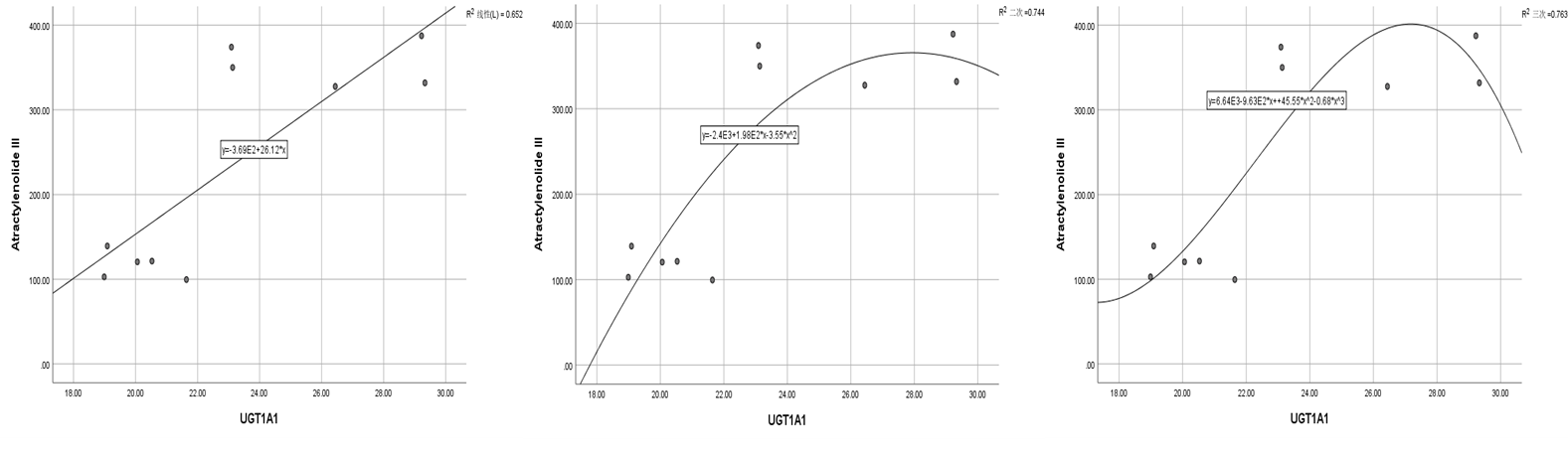
**

**Supplementary Figure 1** The correlation between the expression level of UGT1A1 and the AUC of Atractylenolide III (*p*<0.05)

**
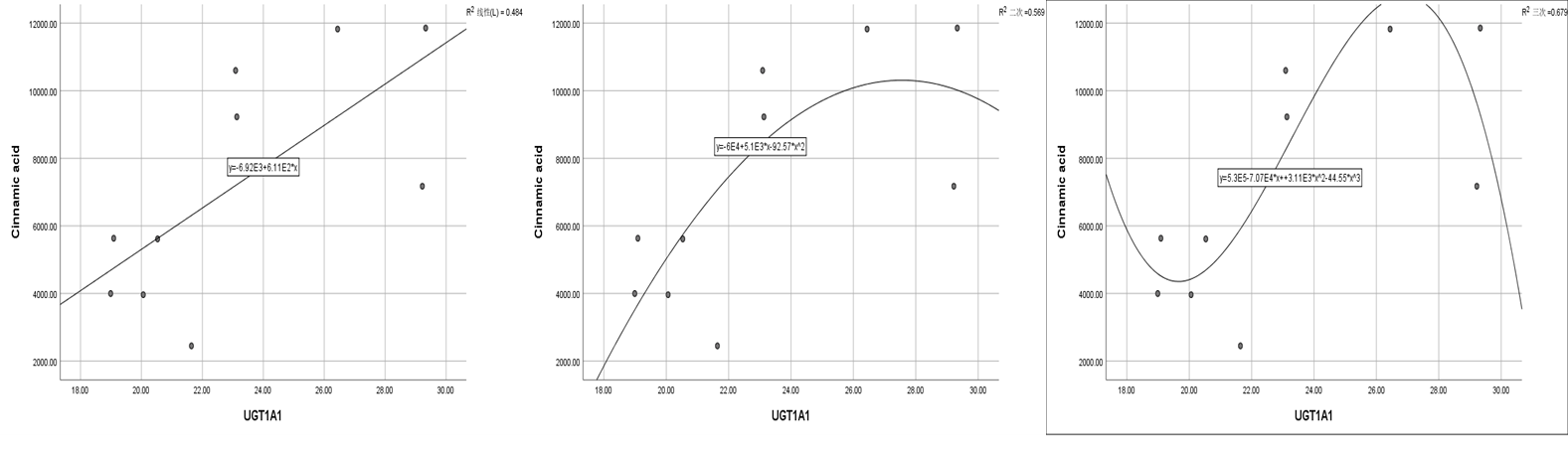
**

**Supplementary Figure 2** The correlation between the expression level of UGT1A1 and the AUC of Cinnamic acid (*p*<0.05)


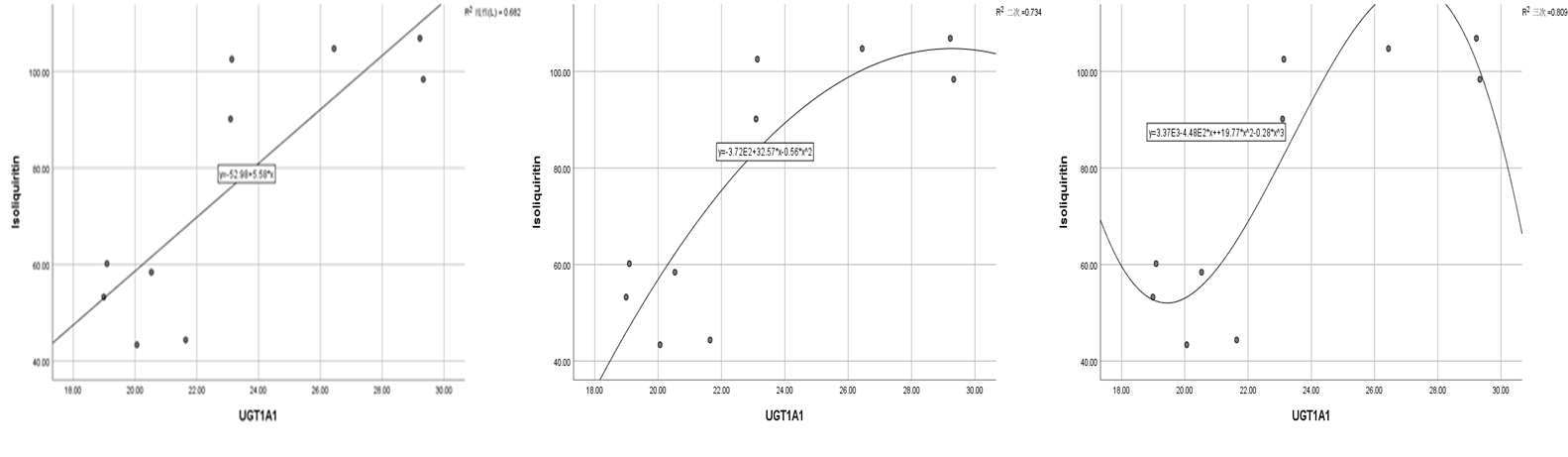


**Supplementary Figure 3** The correlation between the expression level of UGT1A1 and the AUC of Isoliquiritin (*p*<0.01)


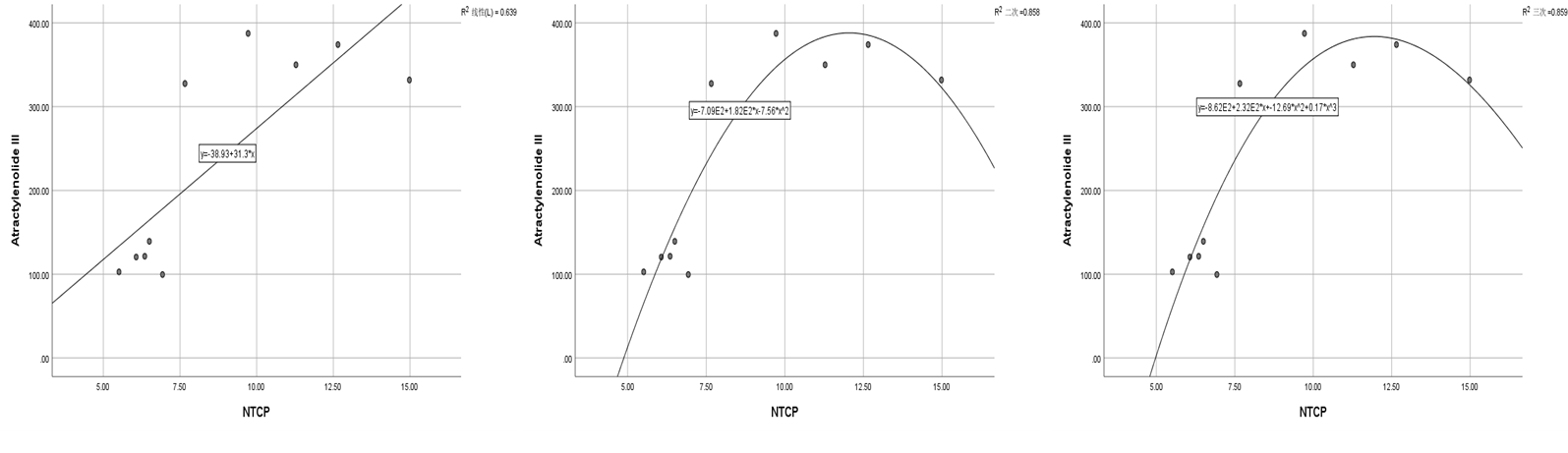


**Supplementary Figure 4** The correlation between the expression level of NTCP and the AUC of Atractylenolide III (*p*<0.01)


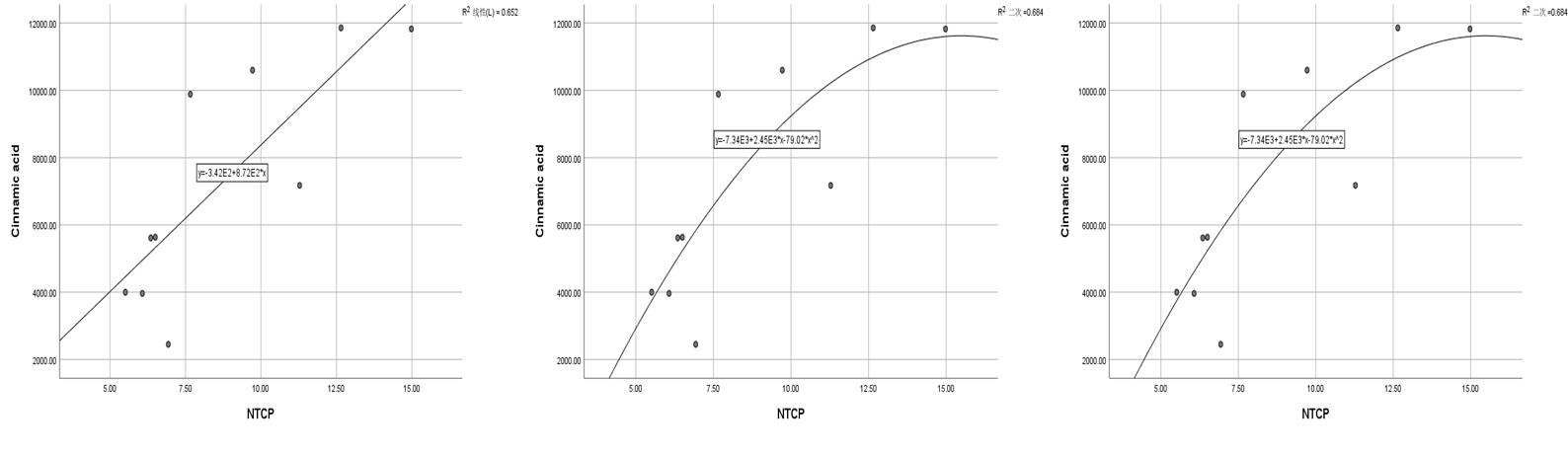


**Supplementary Figure 5** The correlation between the expression level of NTCP and the AUC of Cinnamic acid (*p*<0.01)


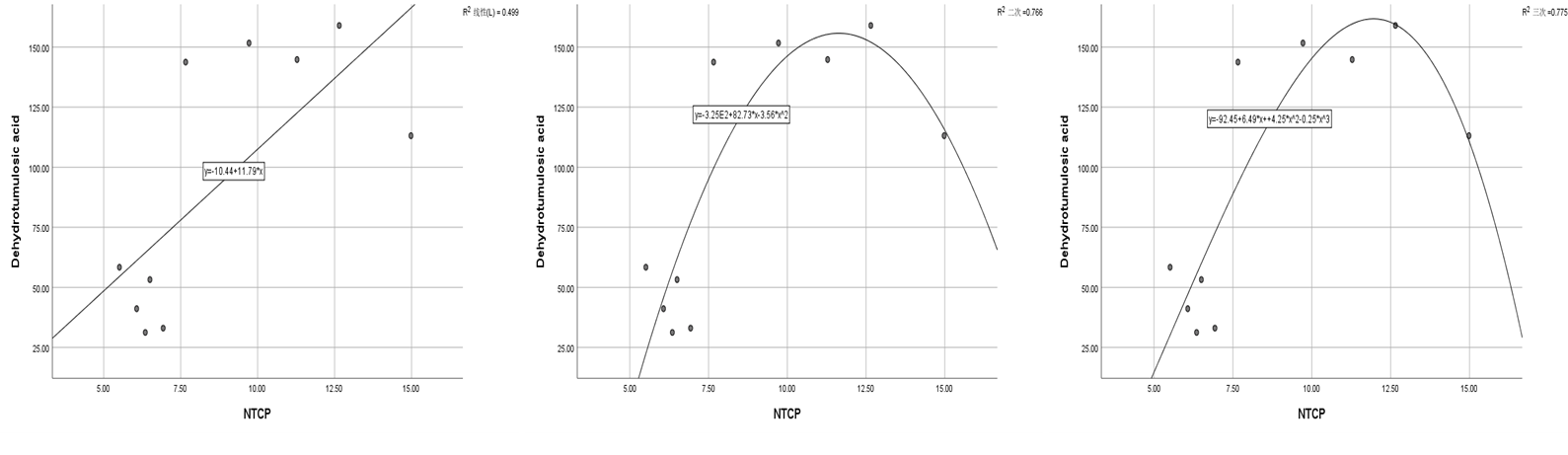


**Supplementary Figure 6** The correlation between the expression level of NTCP and the AUC of Dehydrotumulosic acid (*p*<0.05)


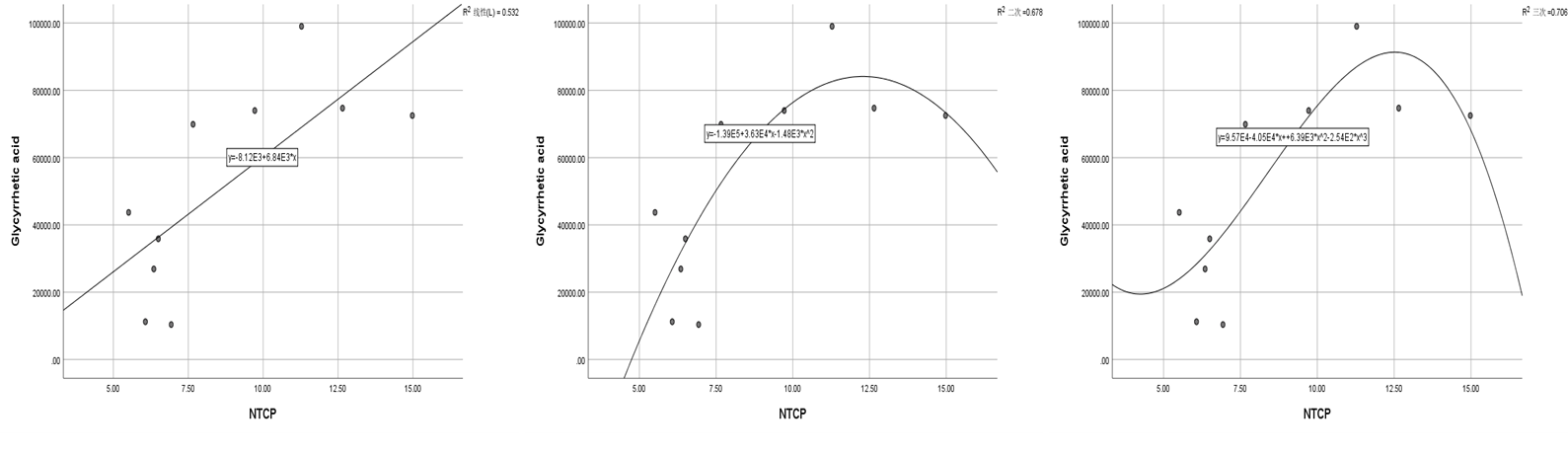


**Supplementary Figure 7** The correlation between the expression level of NTCP and the AUC of Glycyrrhetic acid (*p*<0.05)


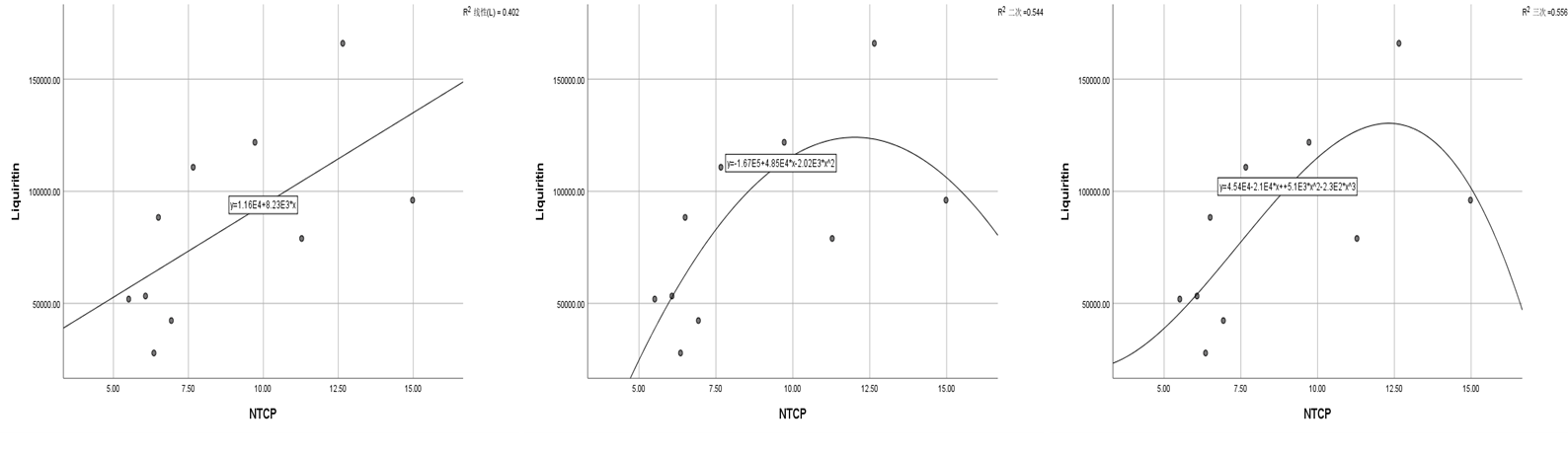


**Supplementary Figure 8** The correlation between the expression level of NTCP and the AUC of Liquiritin (*p*<0.05)


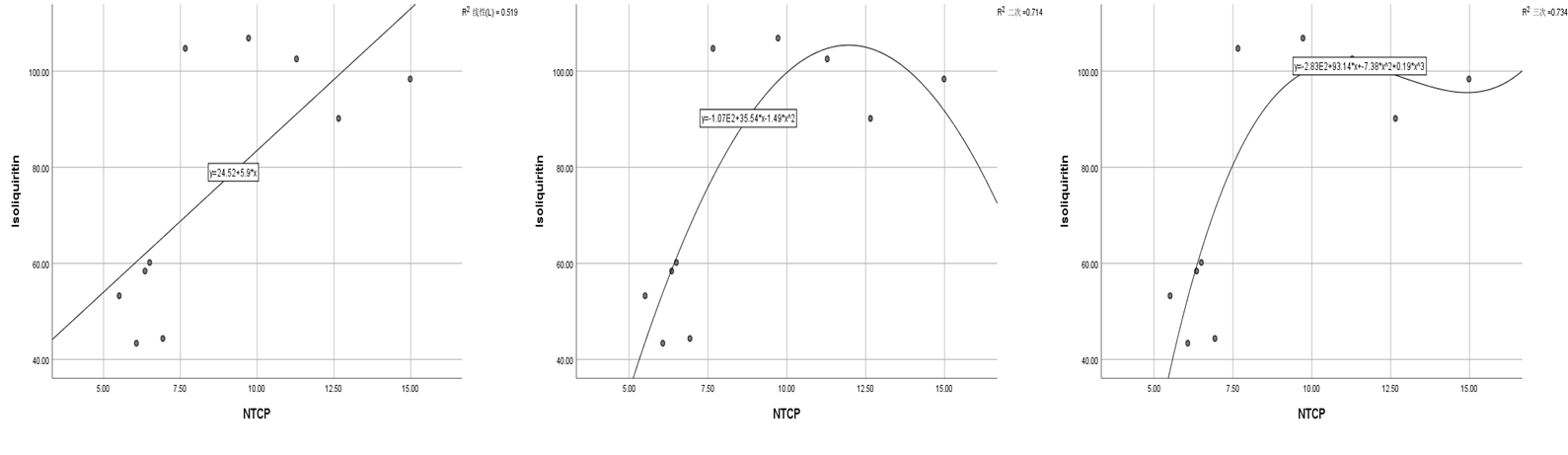


**Supplementary Figure 9** The correlation between the expression level of NTCP and the AUC of Isoliquiritin (*p*<0.05)


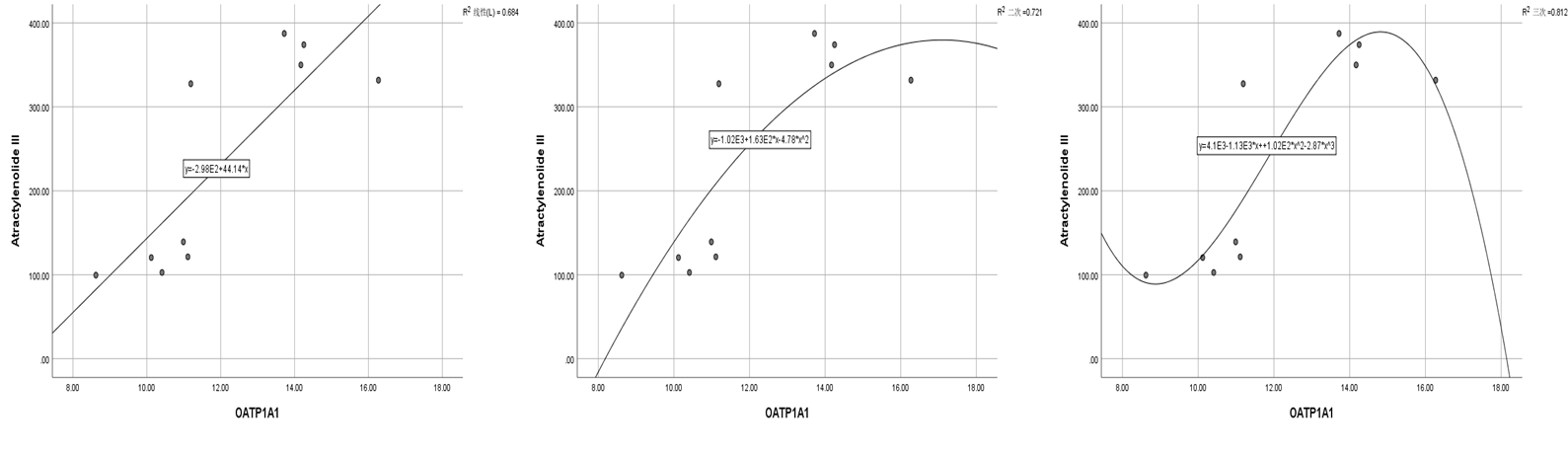


**Supplementary Figure 10** The correlation between the expression level of OATP1A1 and the AUC of Atractylenolide III (*p*<0.01)


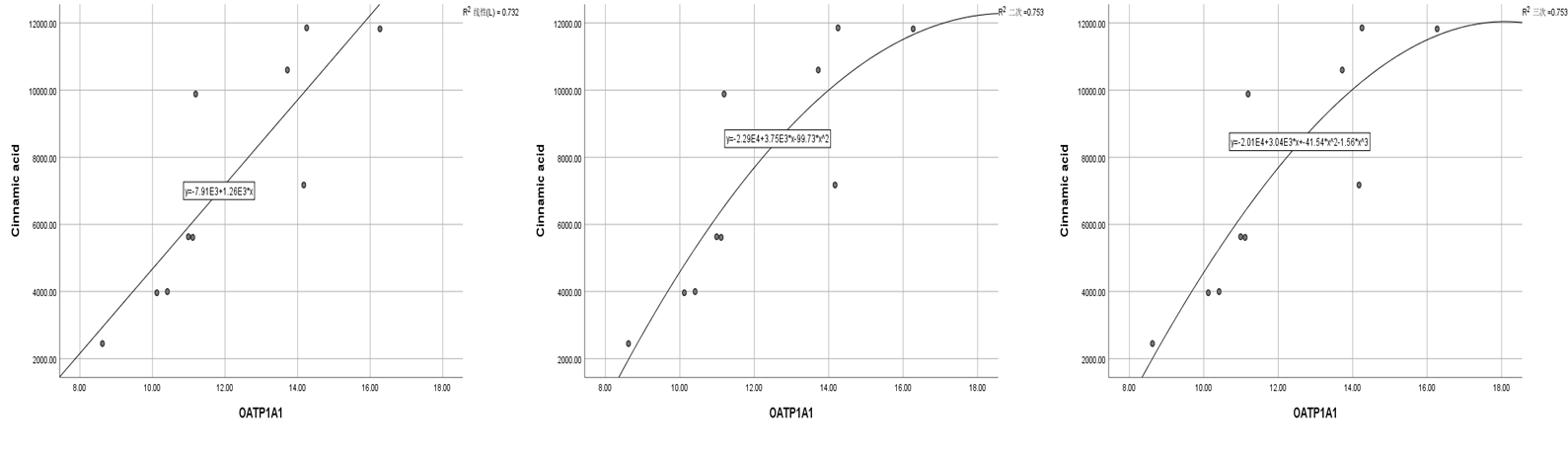


**Supplementary Figure 11** The correlation between the expression level of OATP1A1 and the AUC of Cinnamic acid (*p*<0.01)


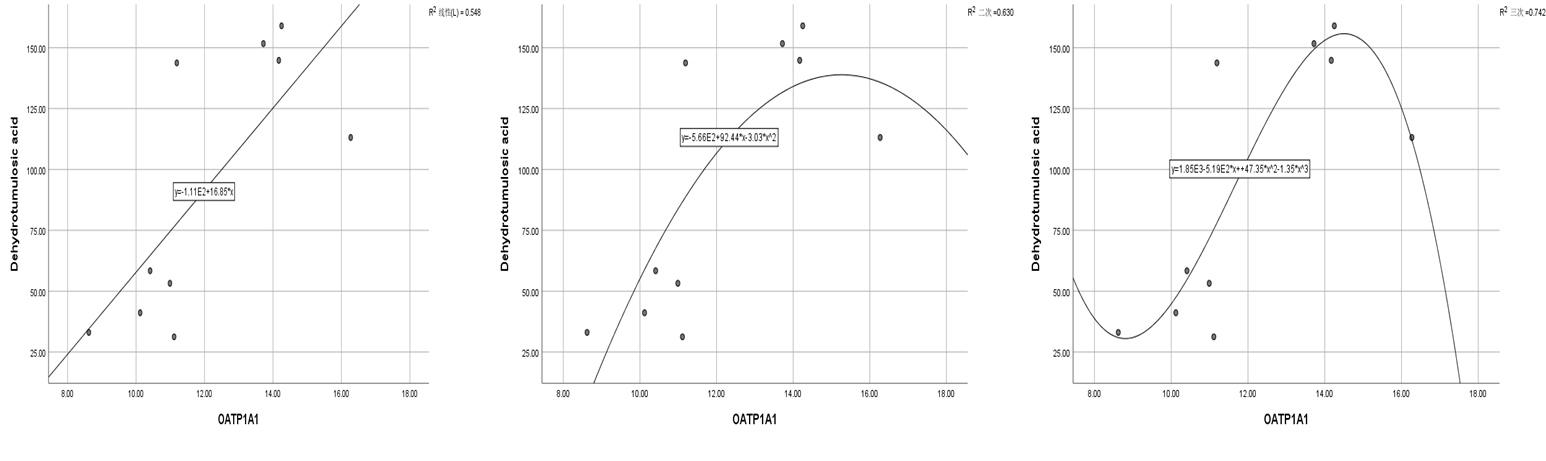


**Supplementary Figure 12** The correlation between the expression level of OATP1A1 and the AUC of Dehydrotumulosic acid (*p*<0.05)


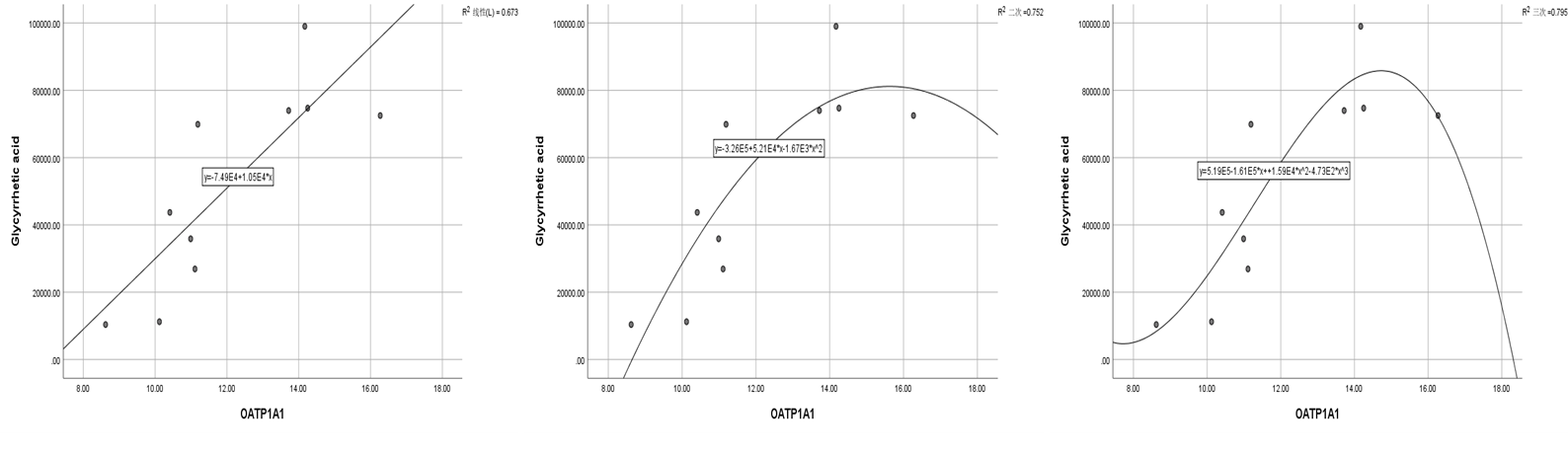


**Supplementary Figure 13** The correlation between the expression level of OATP1A1 and the AUC of Glycyrrhetic acid (*p*<0.01)


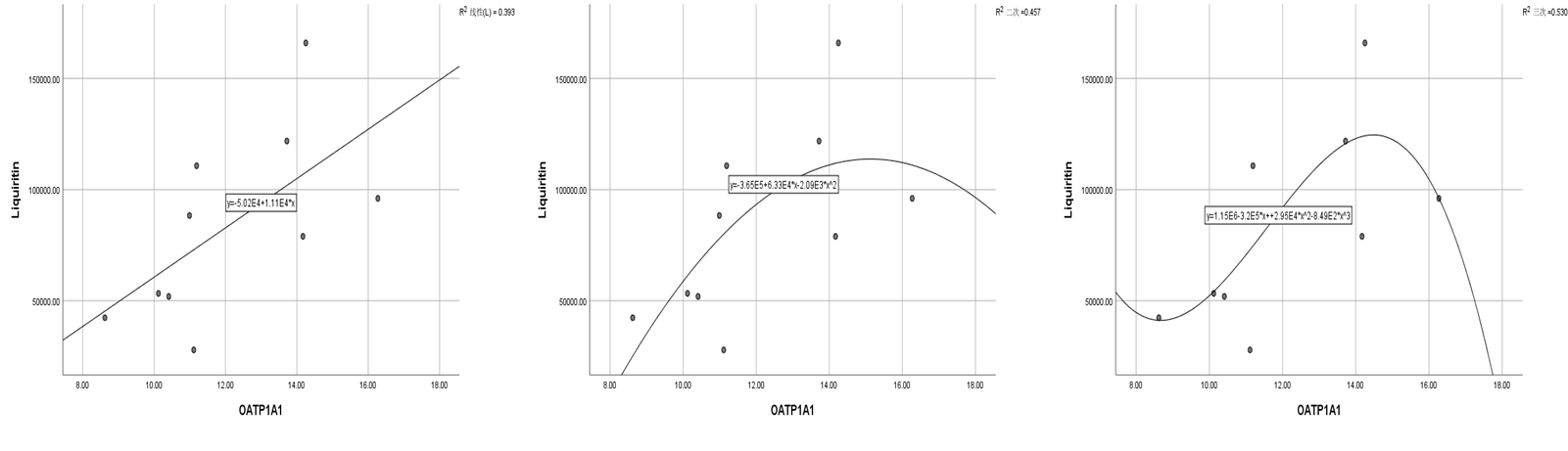


**Supplementary Figure 14** The correlation between the expression level of OATP1A1 and the AUC of Liquiritin (*p*<0.05)


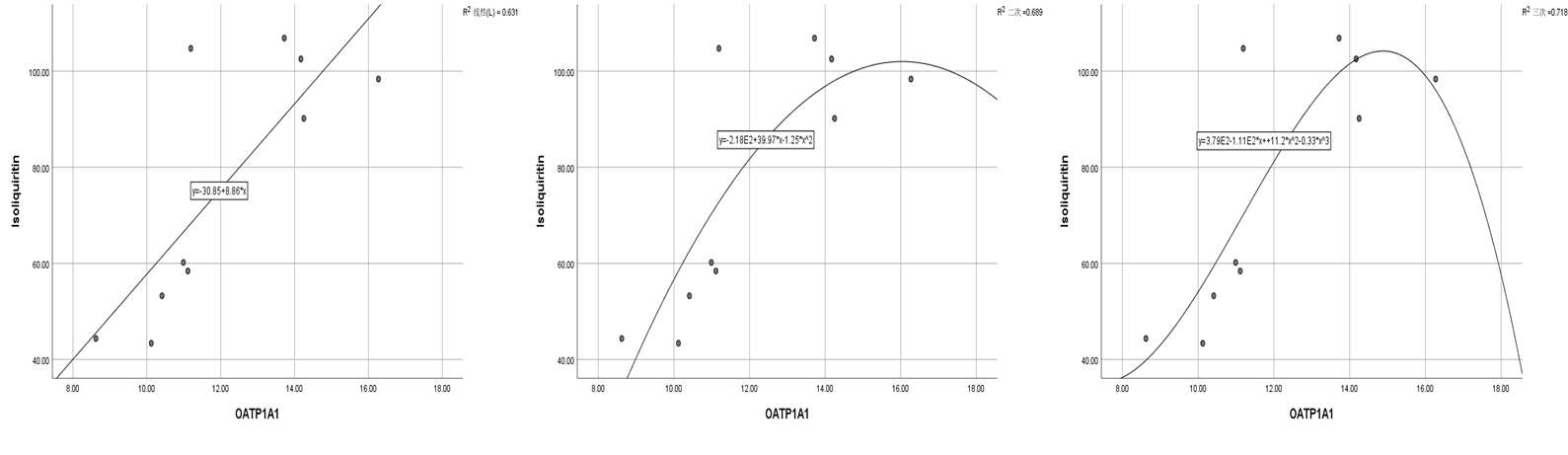


**Supplementary Figure 15** The correlation between the expression level of OATP1A1 and the AUC of Isoliquiritin (*p*<0.05)


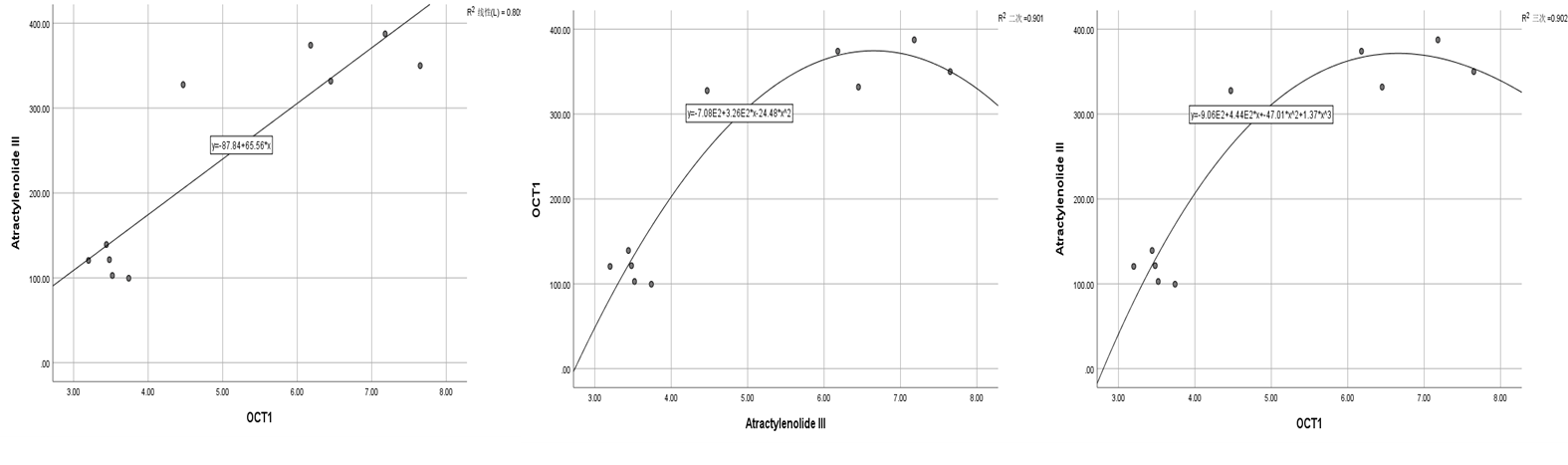


**Supplementary Figure 16** The correlation between the expression level of OCT1 and the AUC of Atractylenolide III (*p*<0.05)


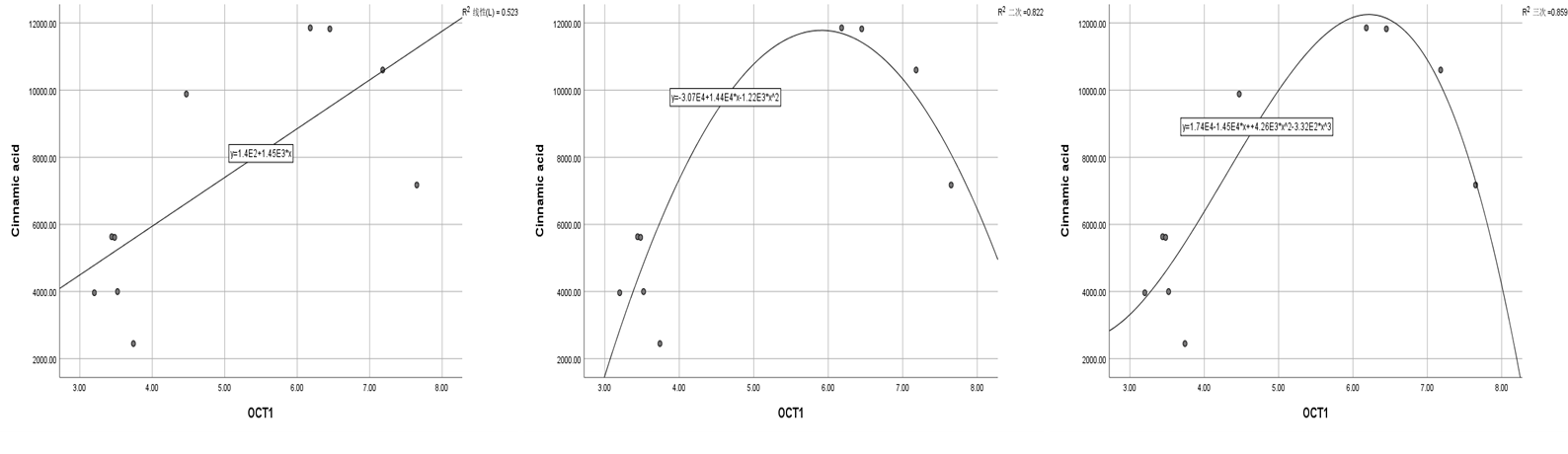


**Supplementary Figure 17** The correlation between the expression level of OCT1 and the AUC of Cinnamic acid (*p*<0.05)


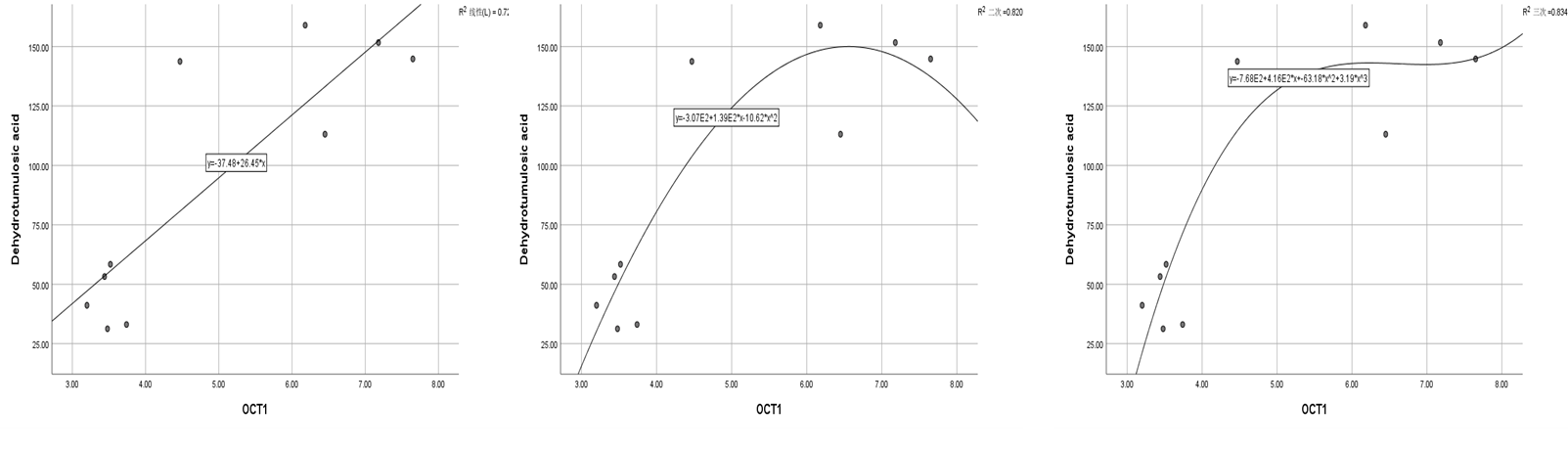


**Supplementary Figure 18** The correlation between the expression level of OCT1 and the AUC of Dehydrotumulosic acid (*p*<0.05)


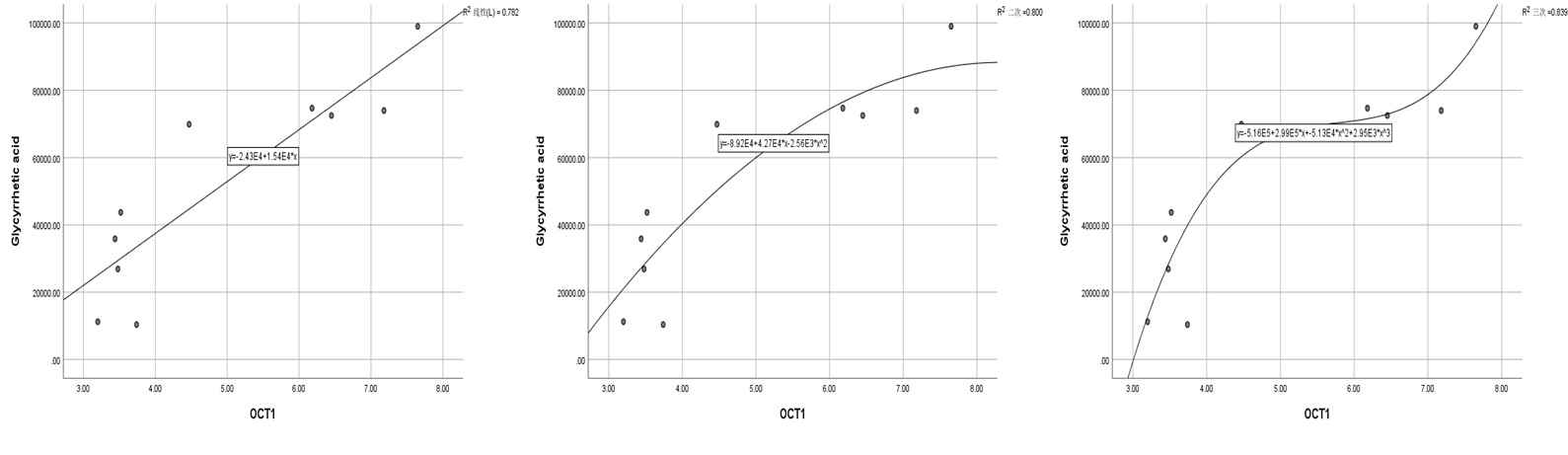


**Supplementary Figure 19** The correlation between the expression level of OCT1 and the AUC of Glycyrrhetic acid (*p*<0.01)


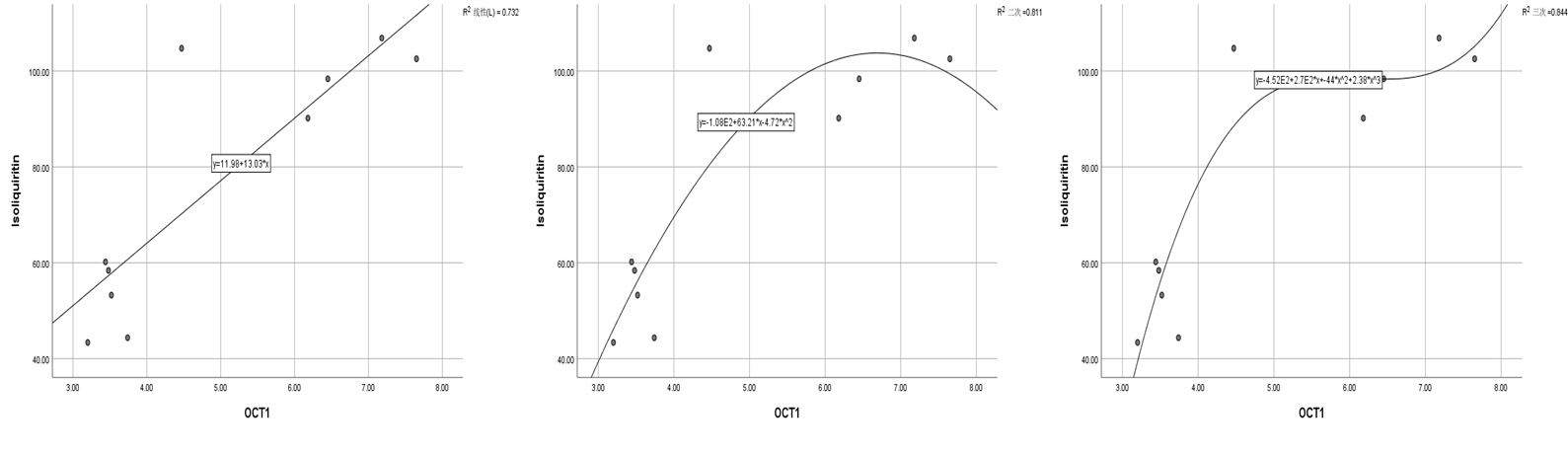


**Supplementary Figure 20** The correlation between the expression level of OCT1 and the AUC of Isoliquiritin (*p*<0.01)


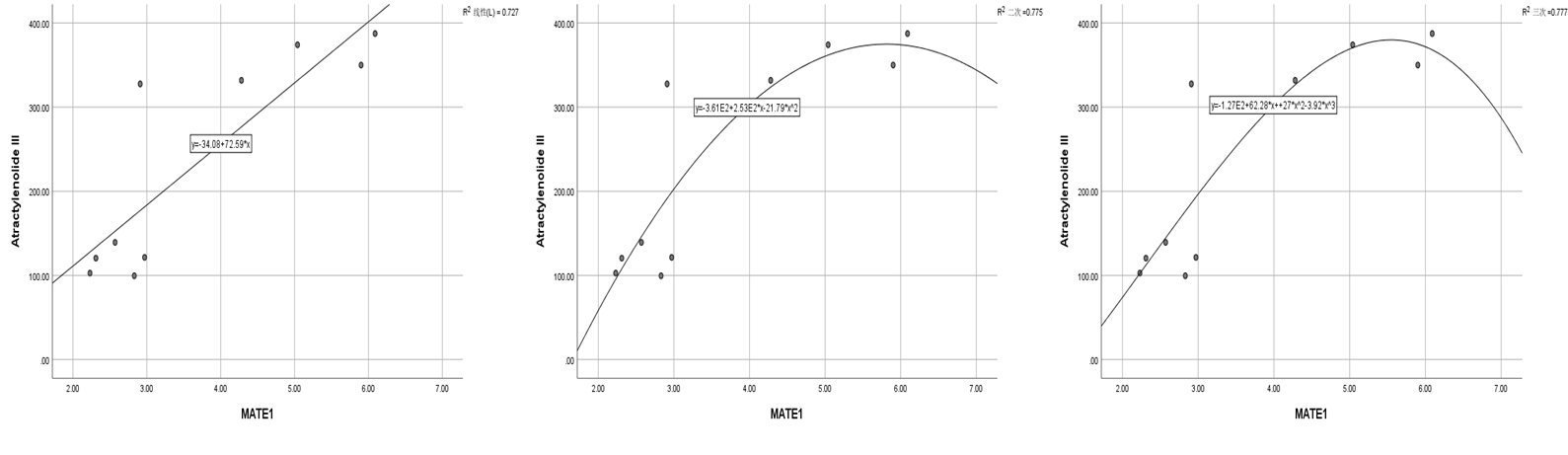


**Supplementary Figure 21** The correlation between the expression level of MATE1 and the AUC of Atractylenolide III (*p*<0.01)


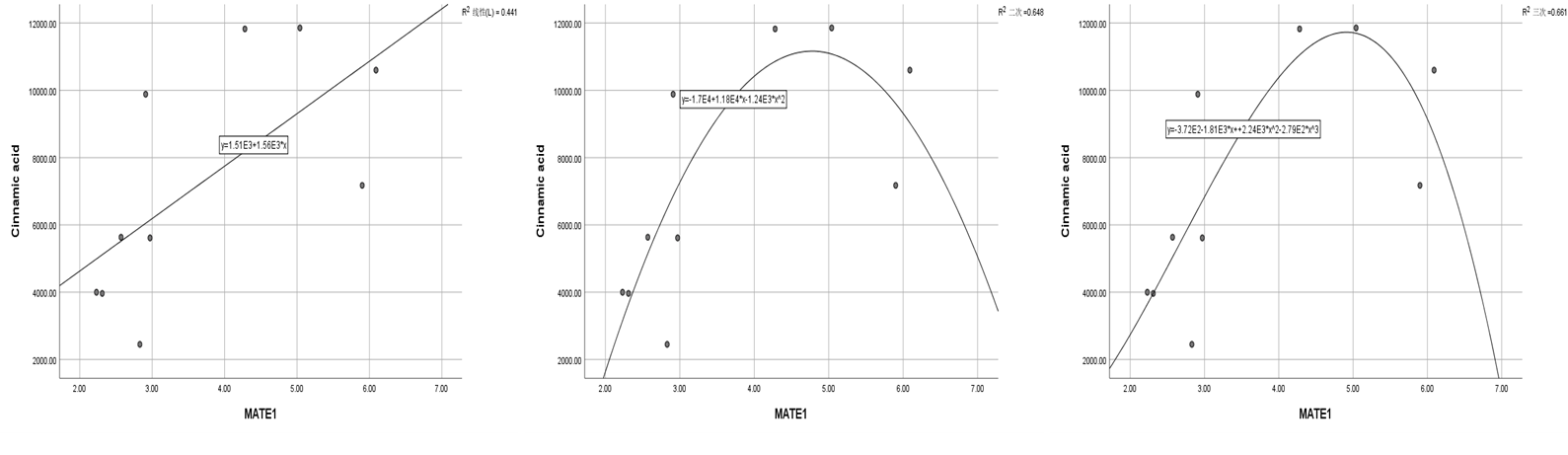


**Supplementary Figure 22** The correlation between the expression level of MATE1 and the AUC of Cinnamic acid (*p*<0.05)


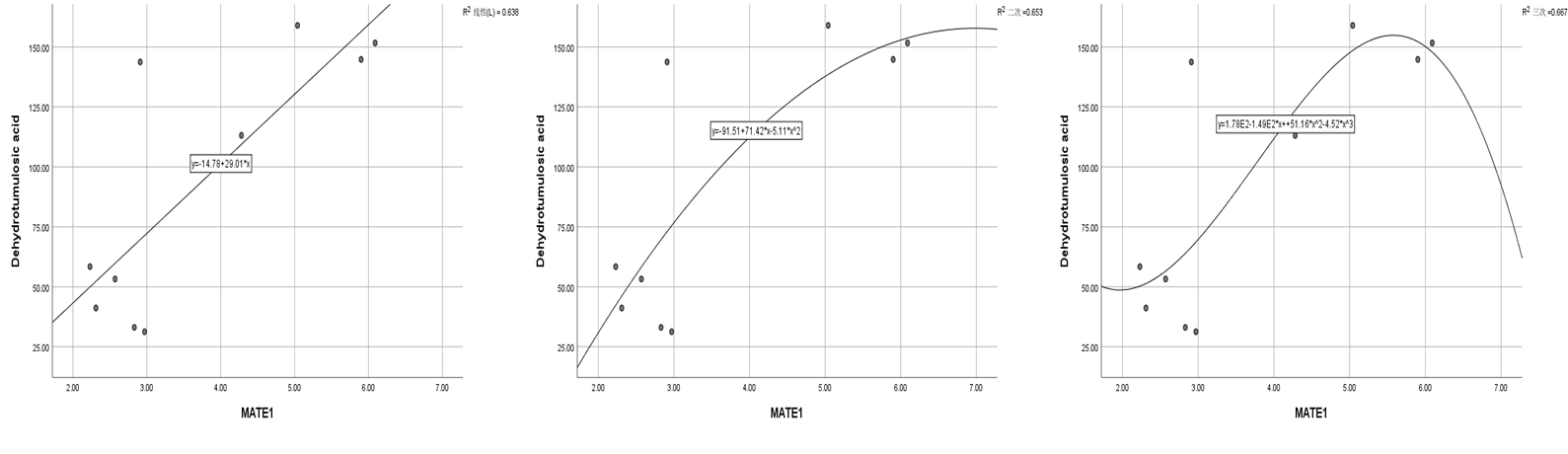


**Supplementary Figure 23** The correlation between the expression level of MATE1 and the AUC of Dehydrotumulosic acid (*p*<0.05)


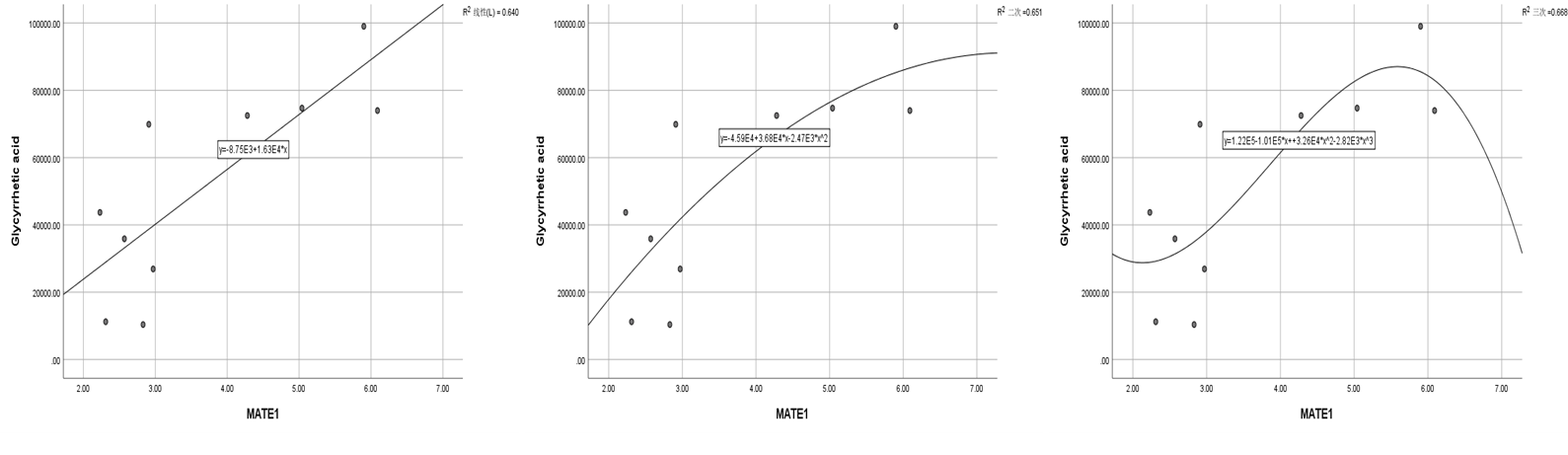


**Supplementary Figure 24** The correlation between the expression level of MATE1 and the AUC of Glycyrrhetic acid (*p*<0.05)


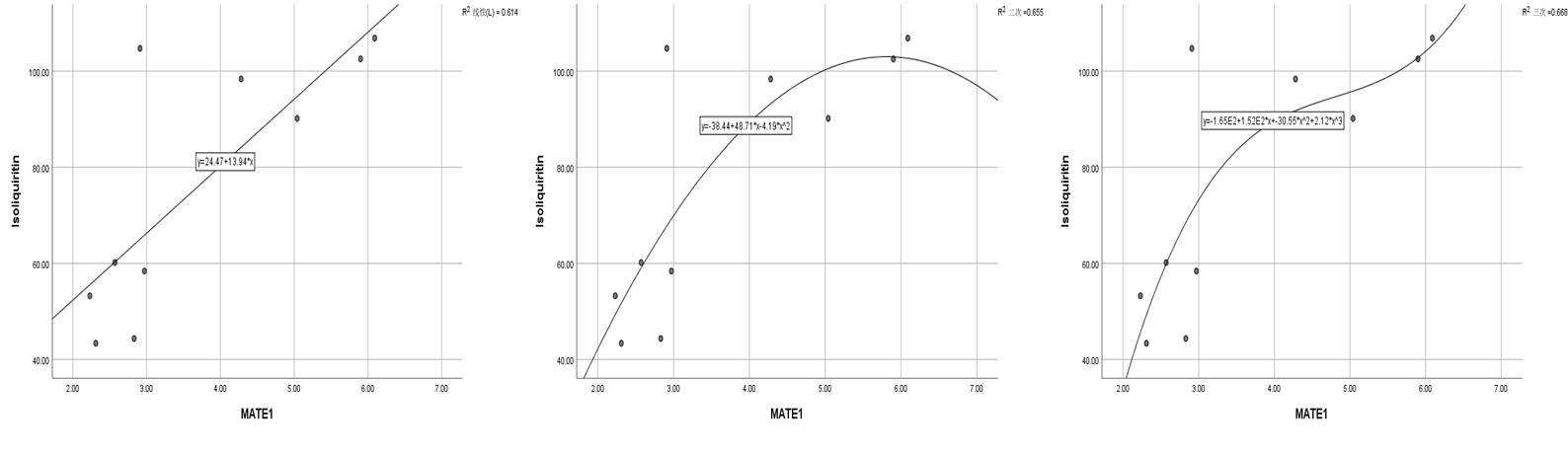


**Supplementary Figure 25** The correlation between the expression level of MATE1 and the AUC of Isoliquiritin (*p*<0.01)


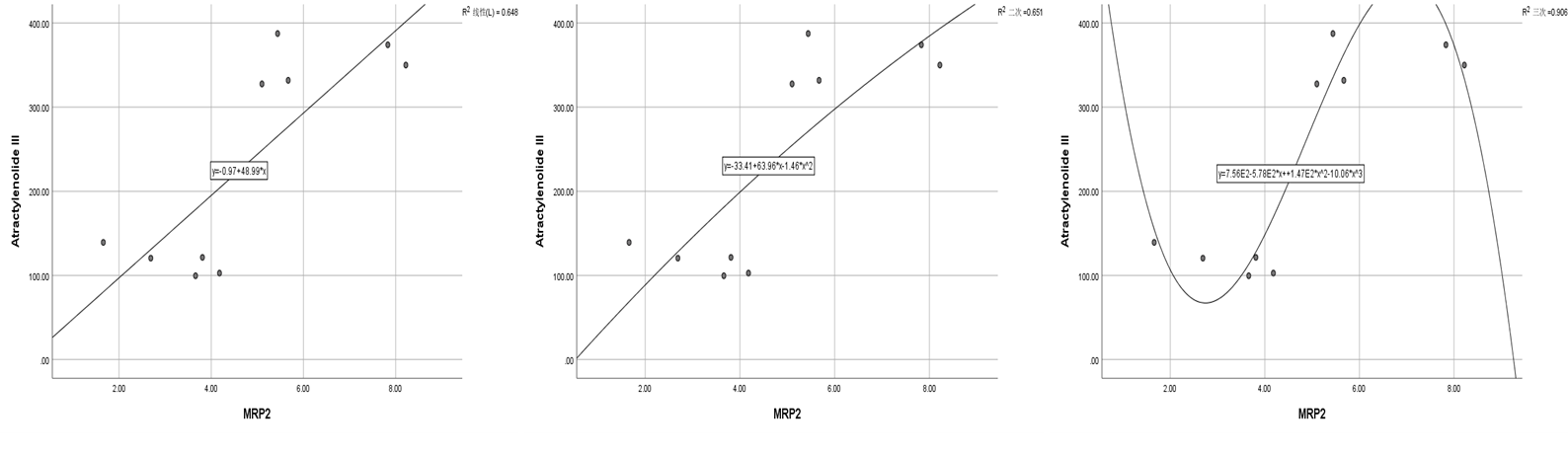


**Supplementary Figure 26** The correlation between the expression level of MRP2 and the AUC of Atractylenolide III (*p*<0.05)


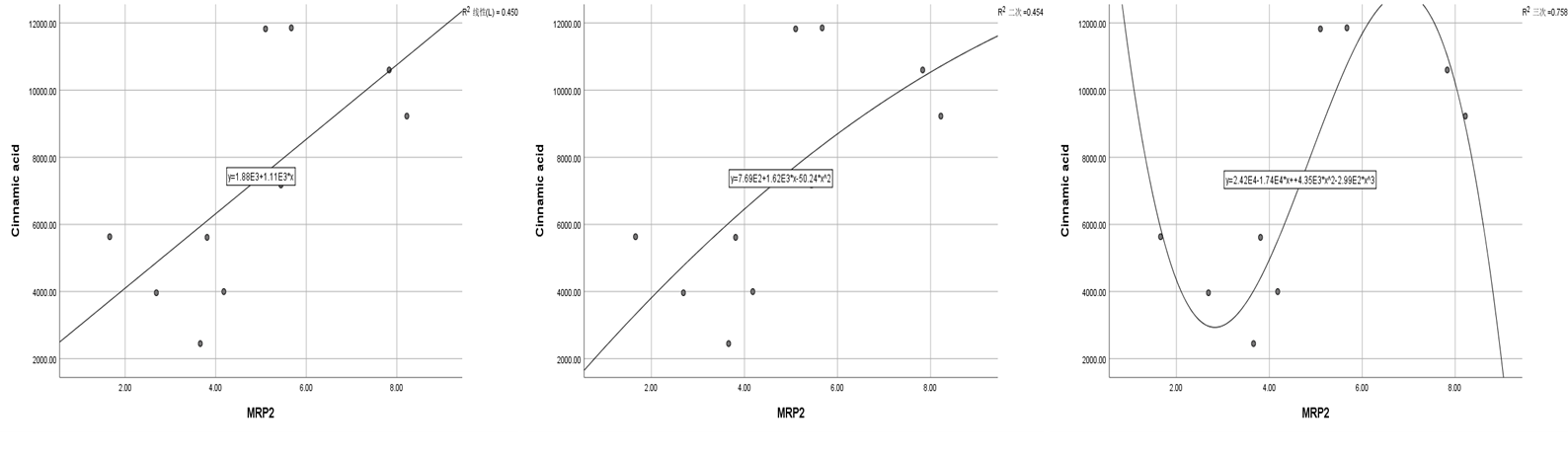


**Supplementary Figure 27** The correlation between the expression level of MRP2 and the AUC of Cinnamic acid (*p*<0.05)


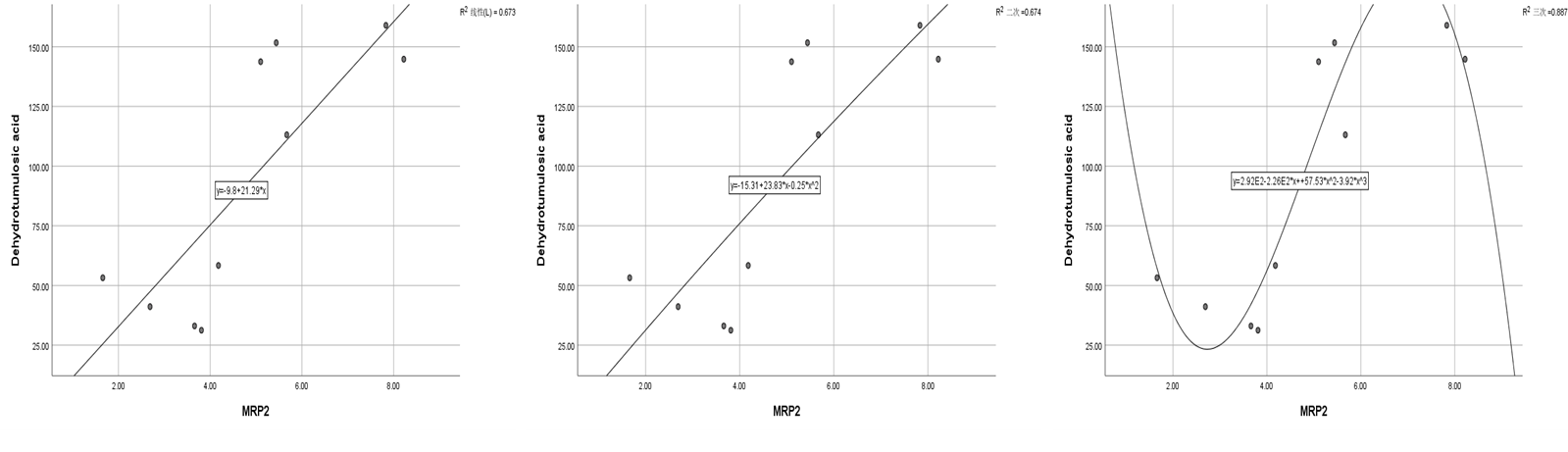


**Supplementary Figure 28** The correlation between the expression level of MRP2 and the AUC of Dehydrotumulosic acid (*p*<0.01)


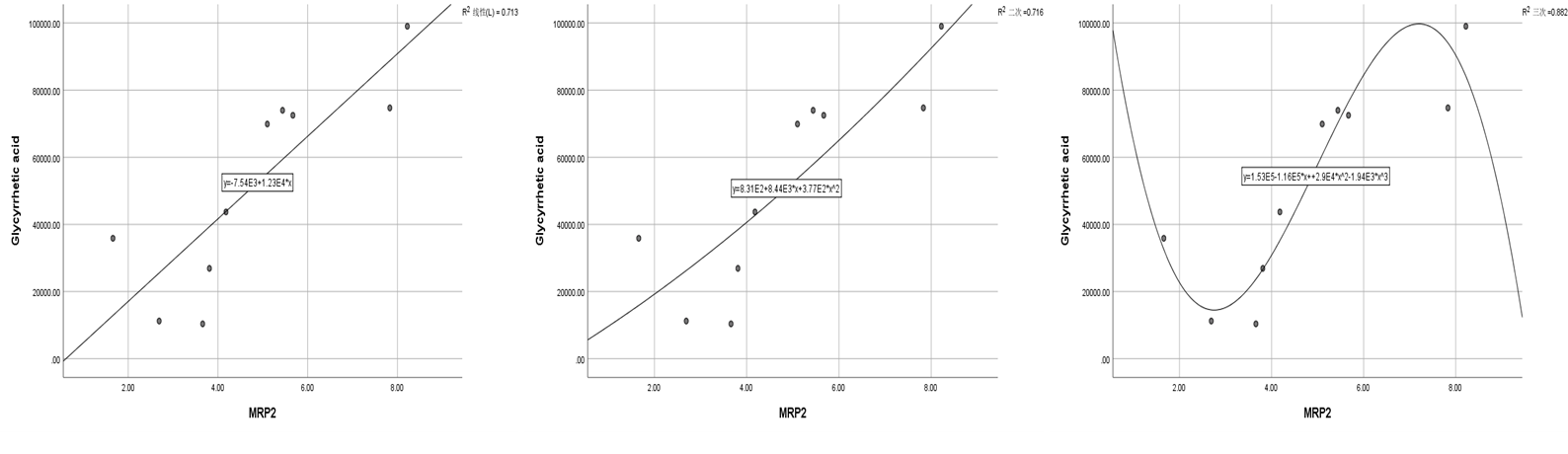


**Supplementary Figure 29** The correlation between the expression level of MRP2 and the AUC of Glycyrrhetic acid (*p*<0.01)


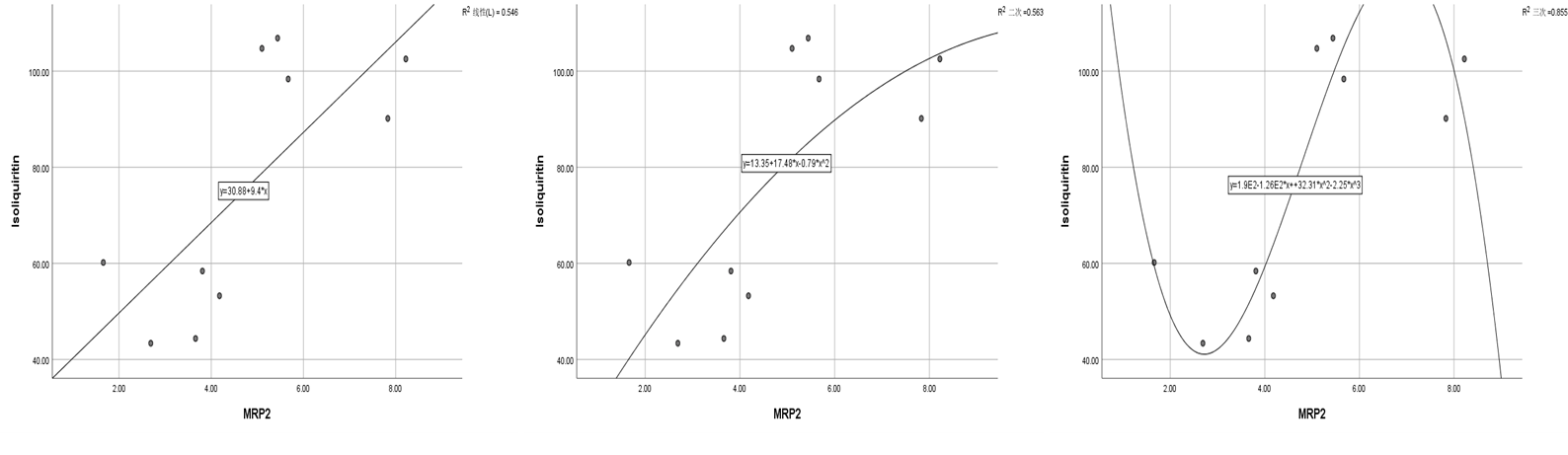


**Supplementary Figure 30** The correlation between the expression level of MRP2 and the AUC of Isoliquiritin (*p*<0.05)


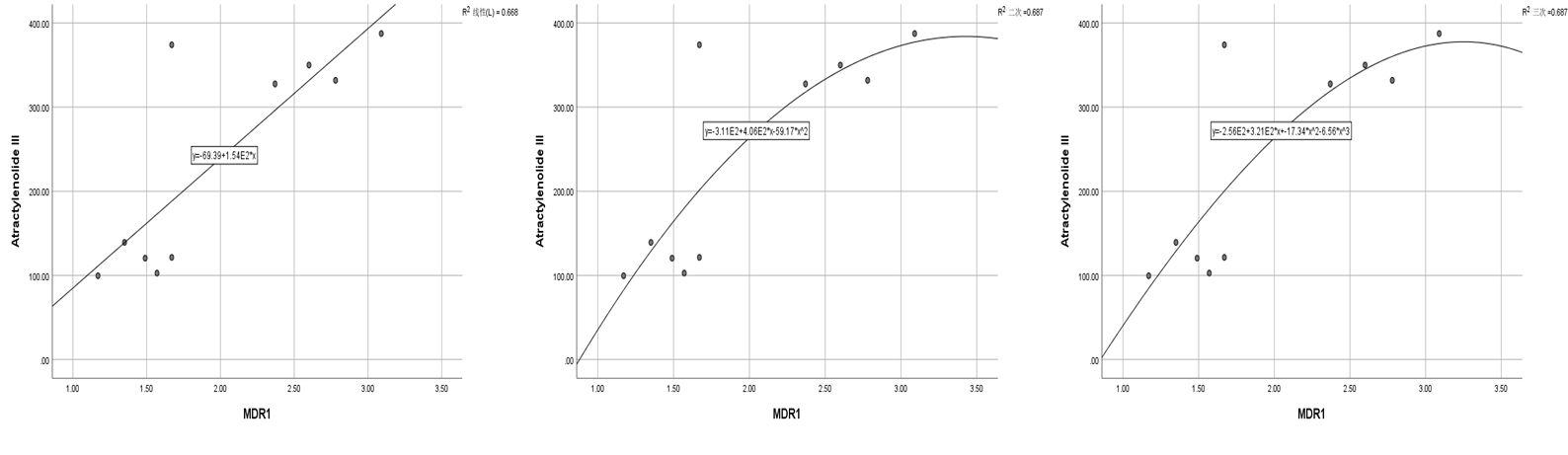


**Supplementary Figure 31** The correlation between the expression level of MDR1 and the AUC of Atractylenolide III (*p*<0.01)


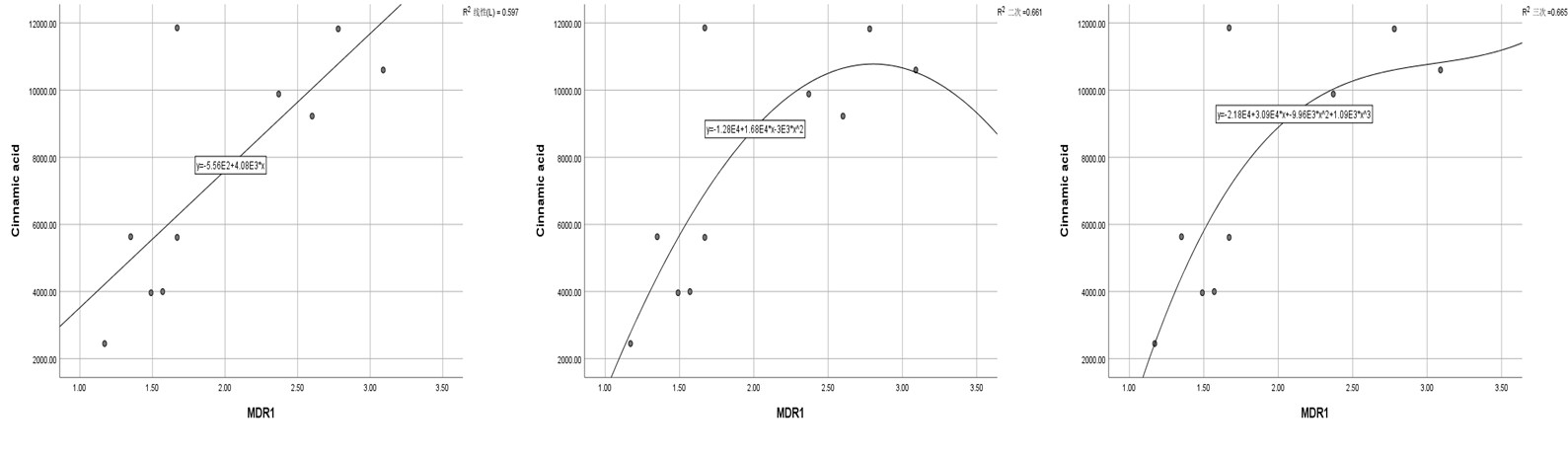


**Supplementary Figure 32** The correlation between the expression level of MDR1 and the AUC of Cinnamic acid (*p*<0.05)


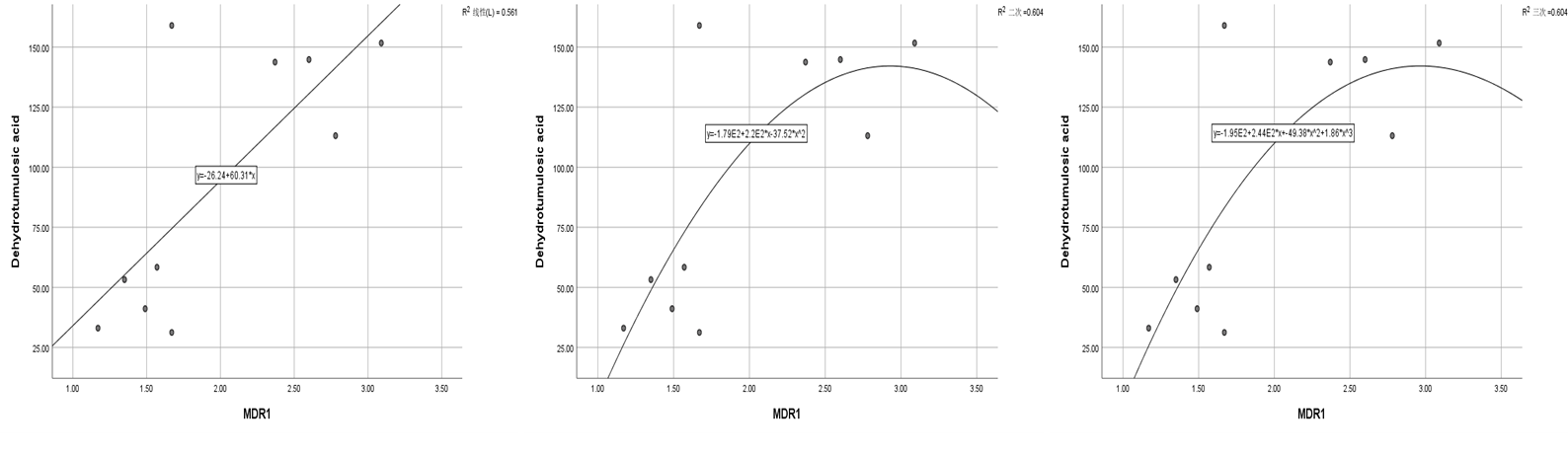


**Supplementary Figure 33** The correlation between the expression level of MDR1 and the AUC of Dehydrotumulosic acid (*p*<0.05)


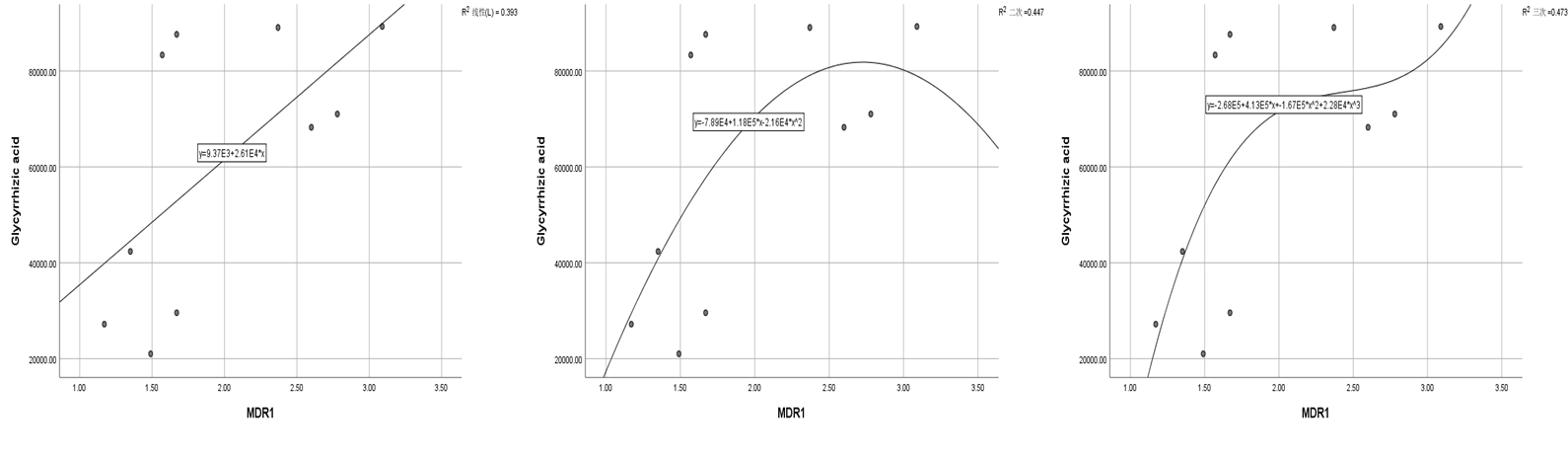


**Supplementary Figure 34** The correlation between the expression level of MDR1 and the AUC of Glycyrrhizic acid (*p*<0.05)


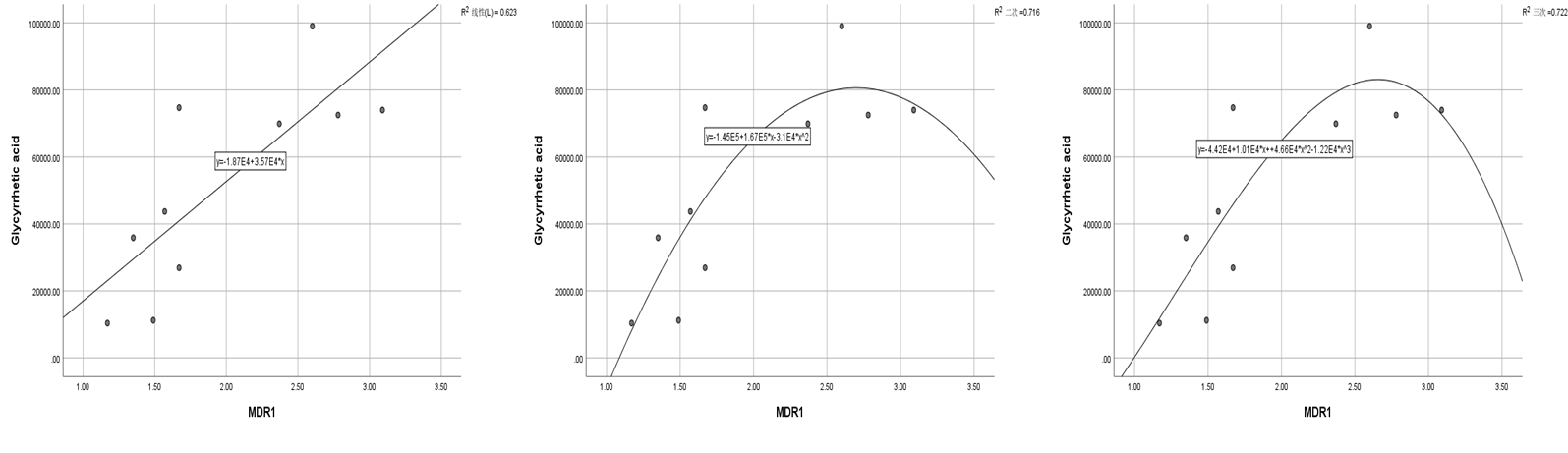


**Supplementary Figure 35** The correlation between the expression level of MDR1 and the AUC of Glycyrrhetic acid (*p*<0.01)


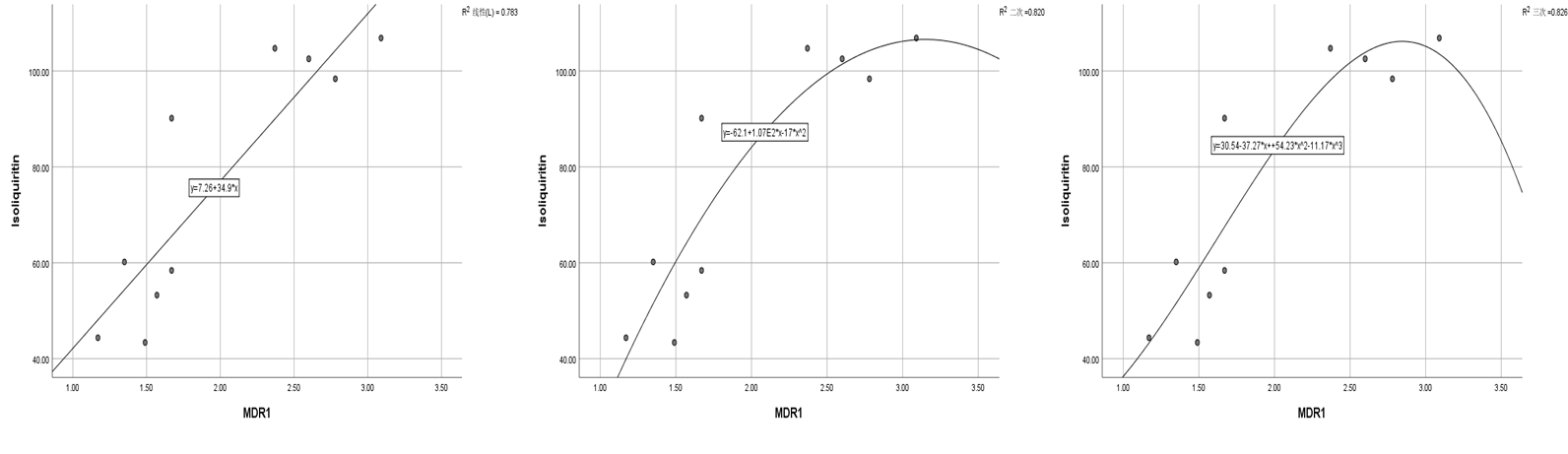


**Supplementary Figure 36** The correlation between the expression level of MDR1 and the AUC of Isoliquiritin (*p*<0.01)


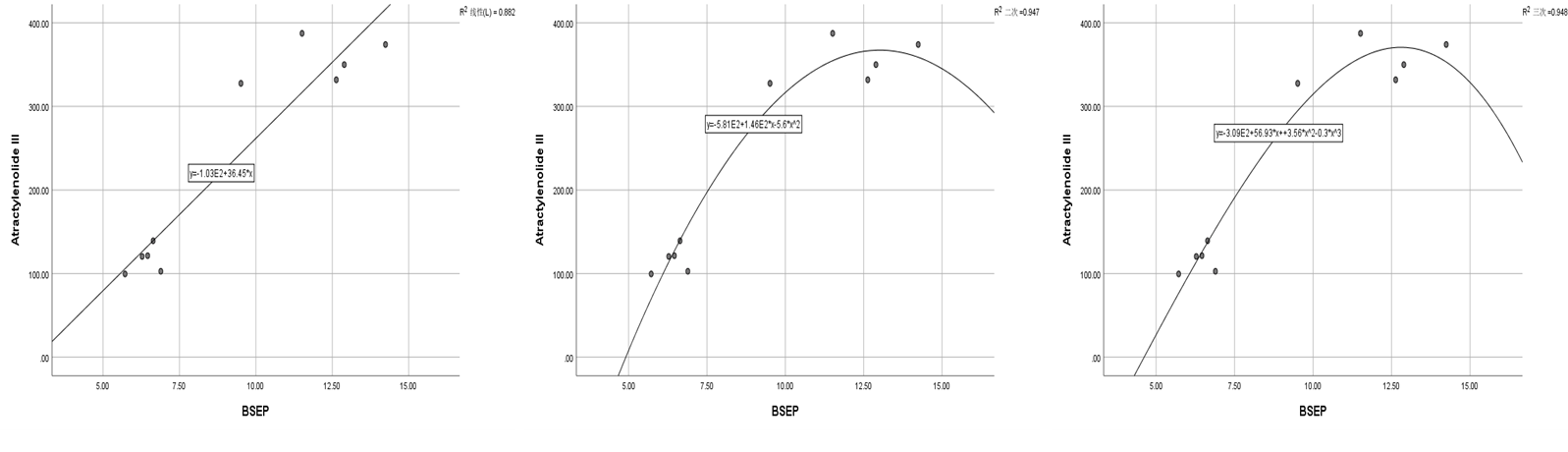


**Supplementary Figure 37** The correlation between the expression level of BSEP and the AUC of Atractylenolide III (*p*<0.01)


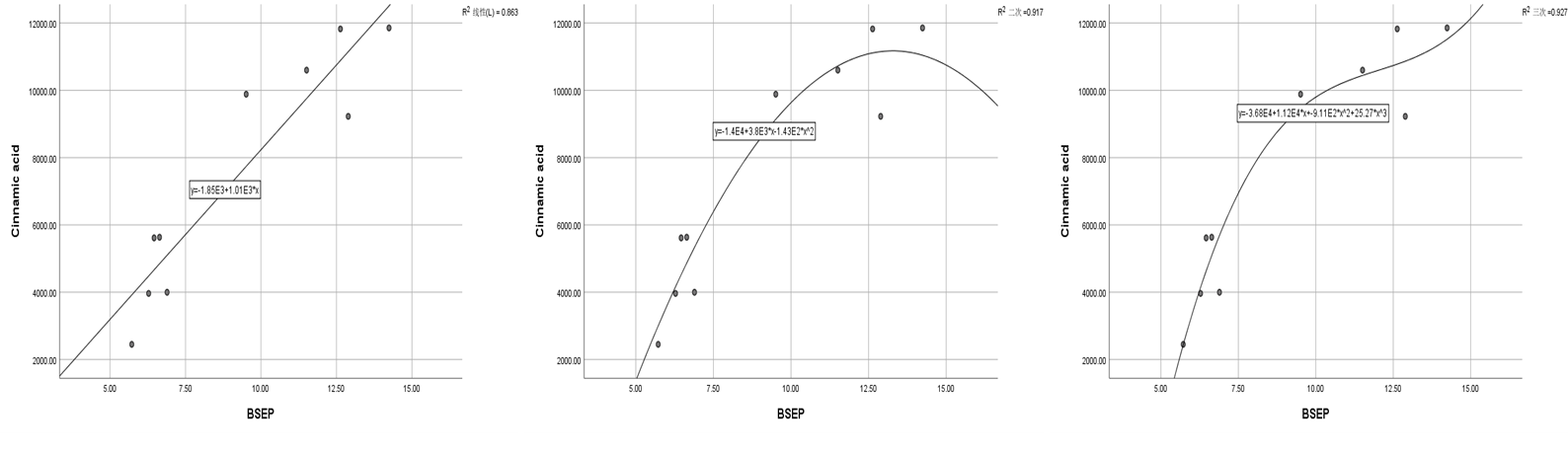


**Supplementary Figure 38** The correlation between the expression level of BSEP and the AUC of Cinnamic acid (*p*<0.01)


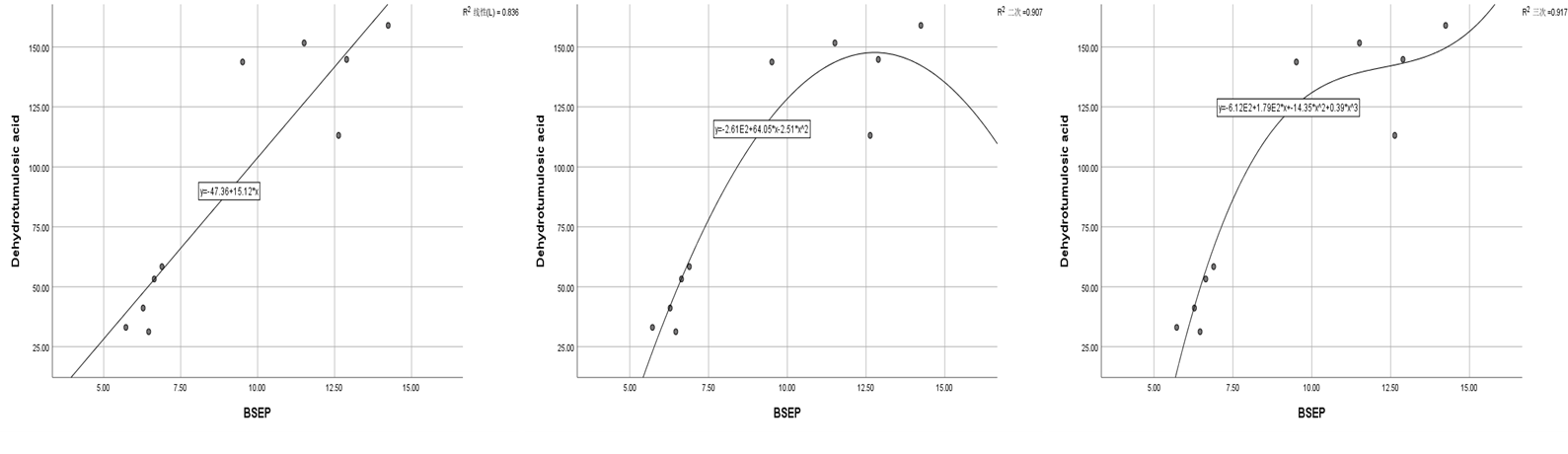


**Supplementary Figure 39** The correlation between the expression level of BSEP and the AUC of Dehydrotumulosic acid (*p*<0.01)


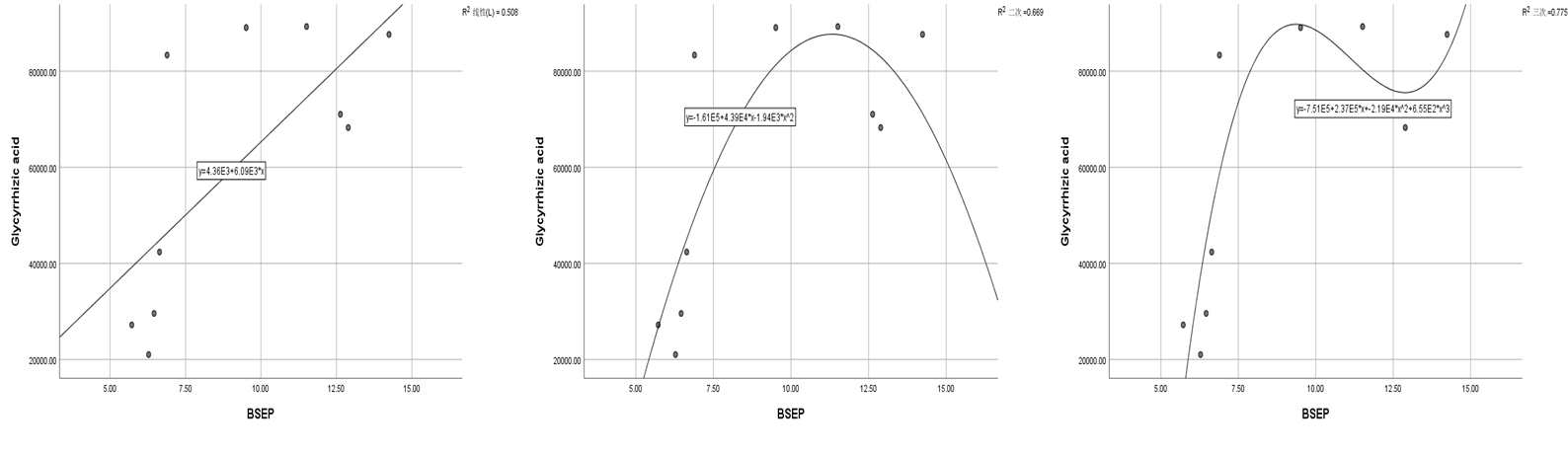


**Supplementary Figure 40** The correlation between the expression level of BSEP and the AUC of Glycyrrhizic acid (*p*<0.05)


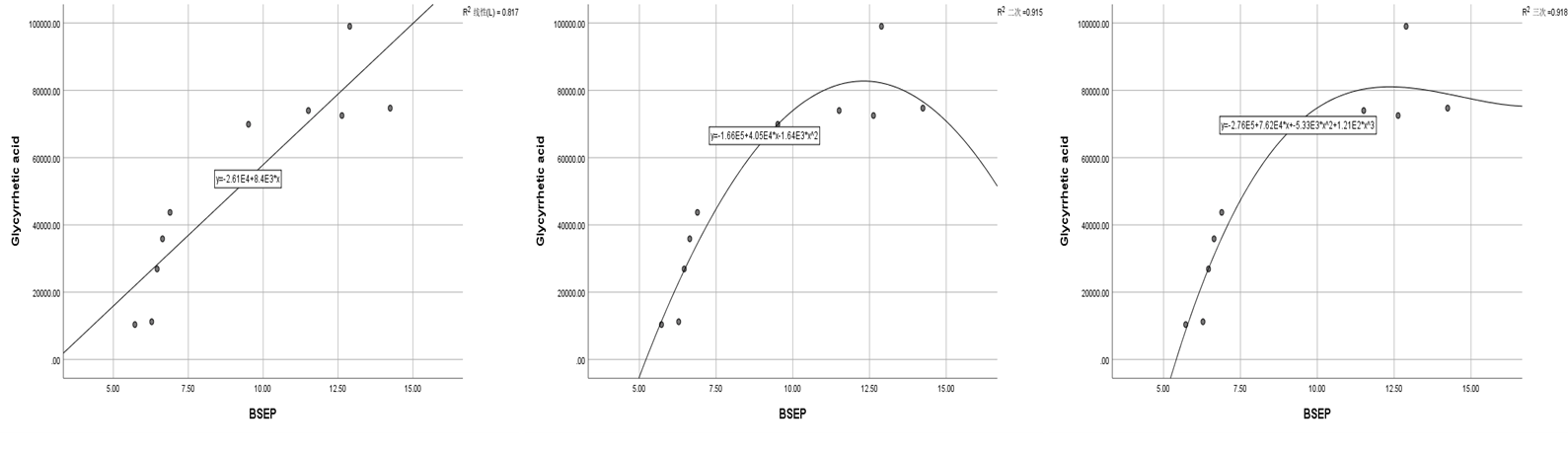


**Supplementary Figure 41** The correlation between the expression level of BSEP and the AUC of Glycyrrhetic acid (*p*<0.01)


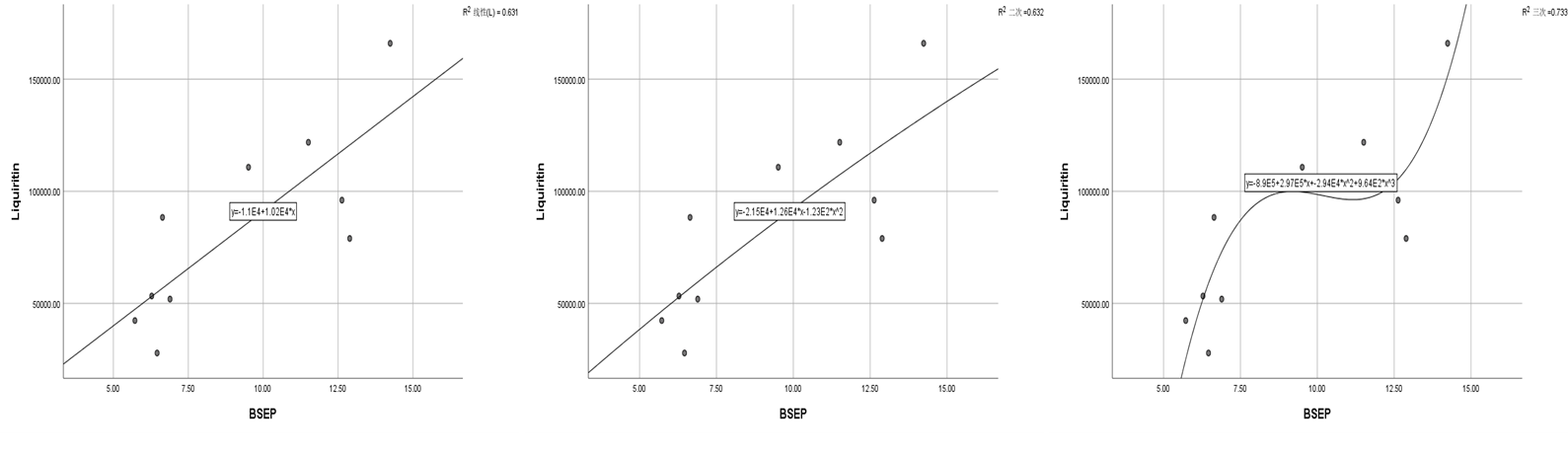


**Supplementary Figure 42** The correlation between the expression level of BSEP and the AUC of Liquiritin (*p*<0.05)


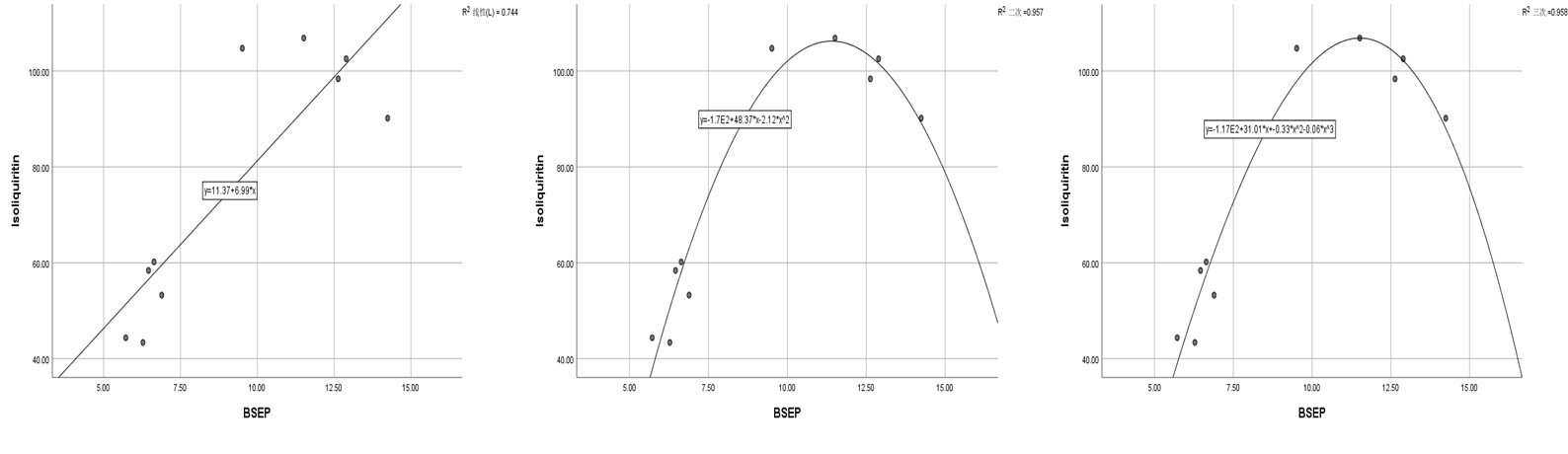


**Supplementary Figure 43** The correlation between the expression level of BSEP and the AUC of Isoliquiritin (*p*<0.05)


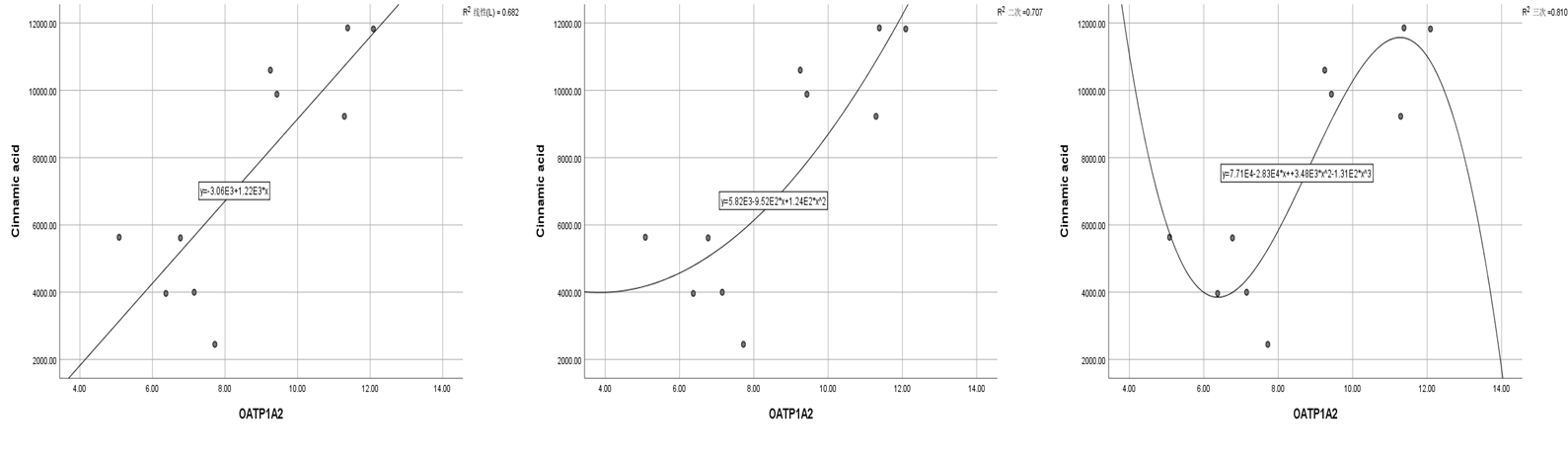


**Supplementary Figure 44** The correlation between the expression level of OATP1A2 and the AUC of Cinnamic acid (*p*<0.05)


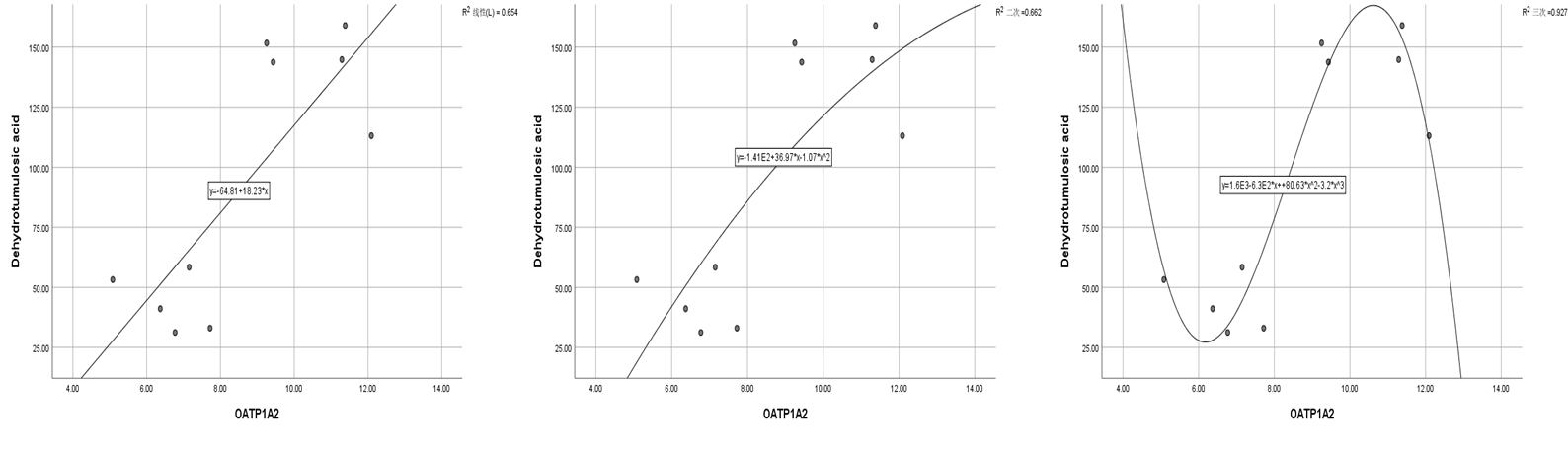


**Supplementary Figure 45** The correlation between the expression level of OATP1A2 and the AUC of Dehydrotumulosic acid (*p*<0.05)


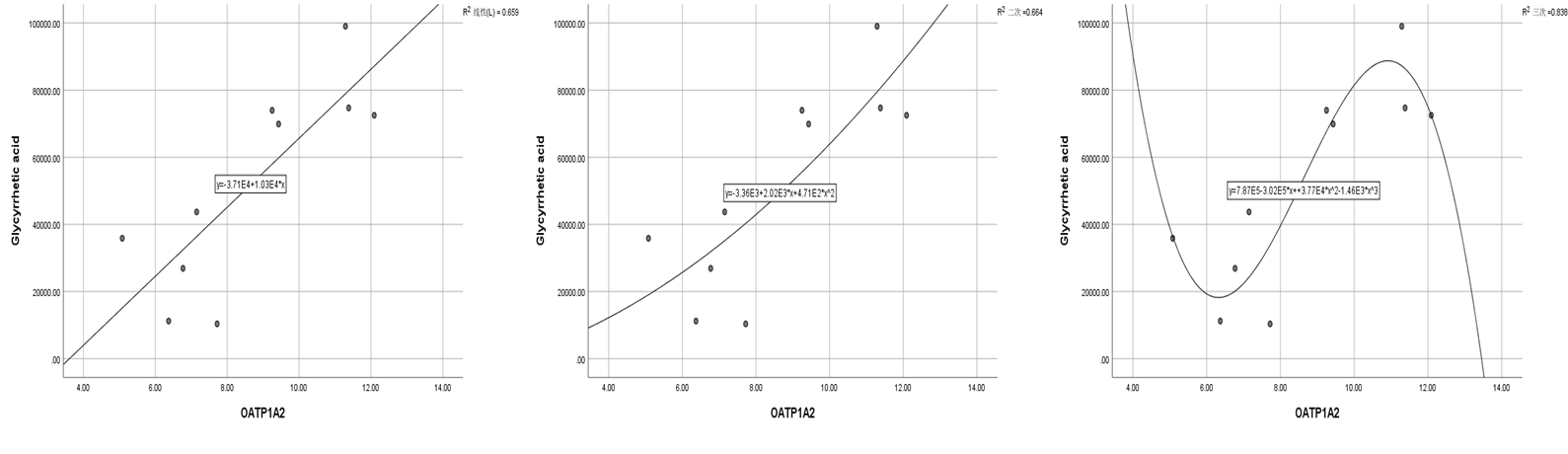


**Supplementary Figure 46** The correlation between the expression level of OATP1A2 and the AUC of Glycyrrhetic acid (*p*<0.05)

**
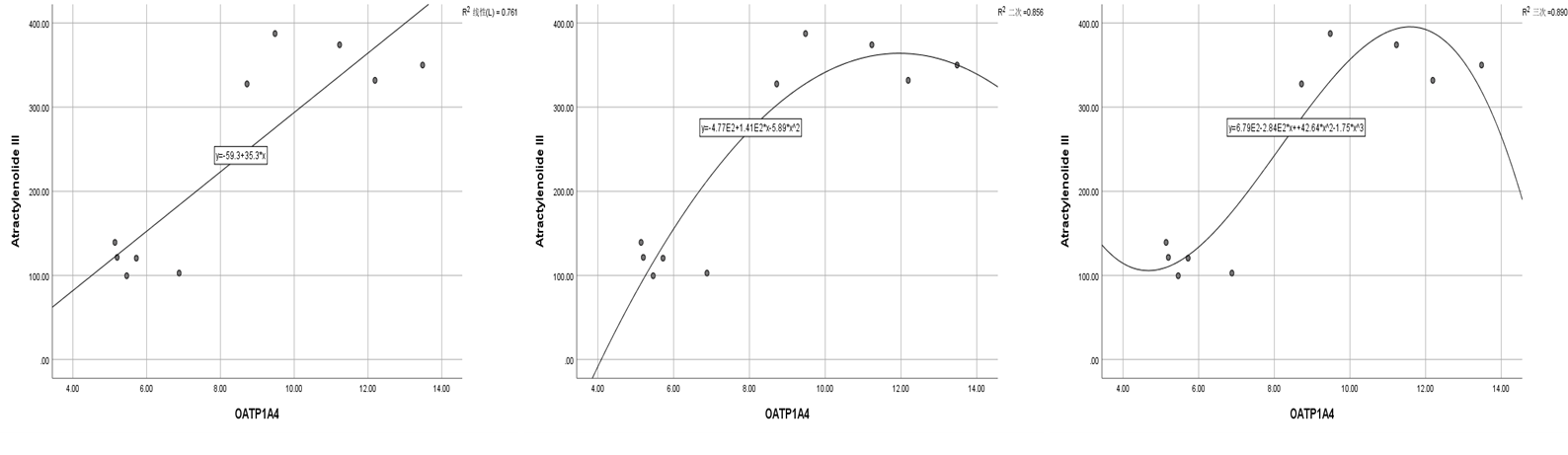
**

**Supplementary Figure 47** The correlation between the expression level of OATP1A4 and the AUC of Atractylenolide III (*p*<0.05)


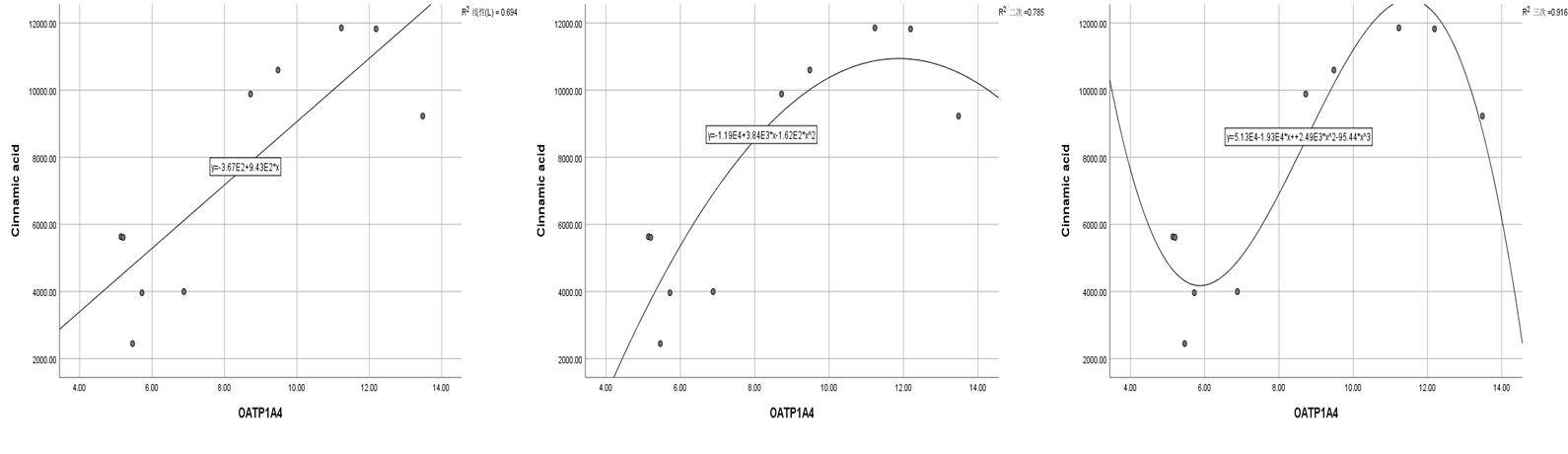


**Supplementary Figure 48** The correlation between the expression level of OATP1A4 and the AUC of Cinnamic acid (*p*<0.05)


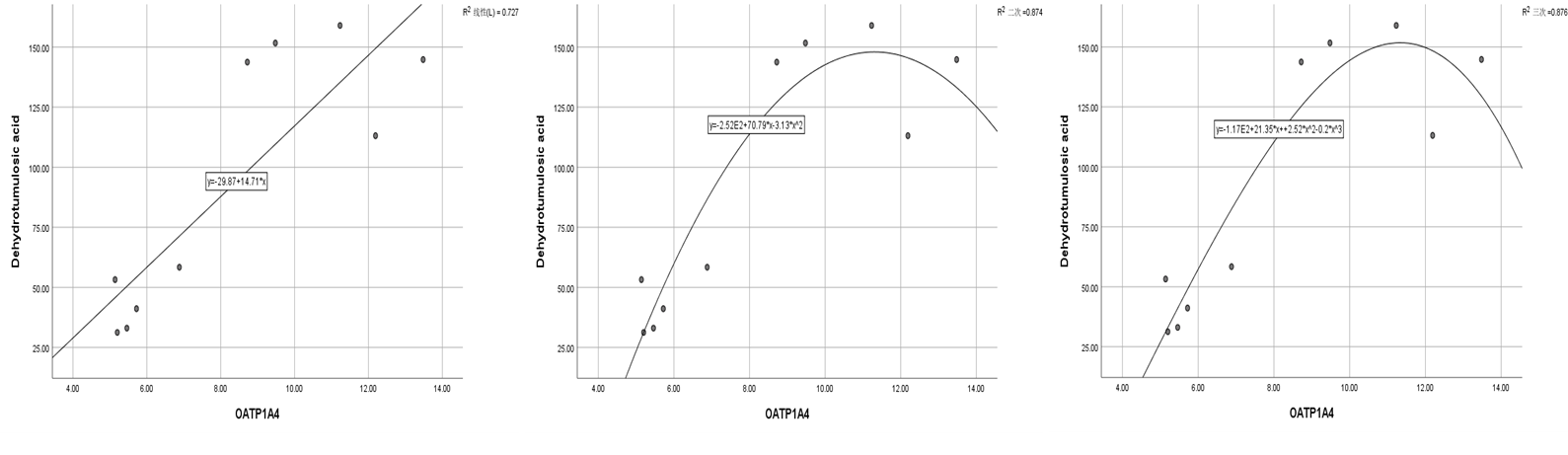


**Supplementary Figure 49** The correlation between the expression level of OATP1A4 and the AUC of Dehydrotumulosic acid (*p*<0.01)


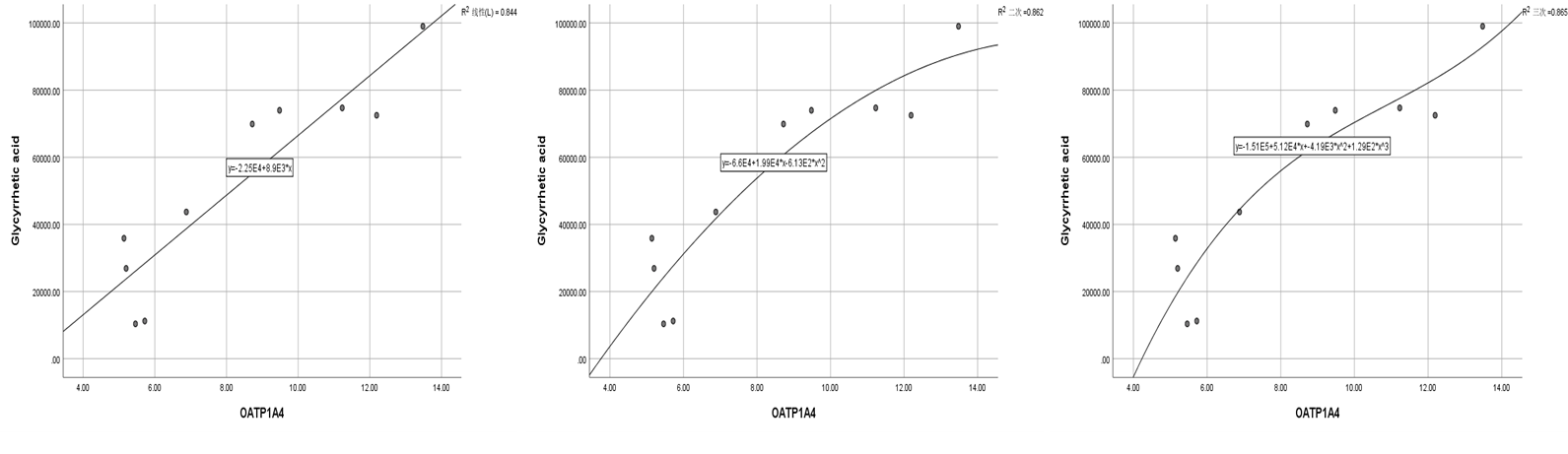


**Supplementary Figure 50** The correlation between the expression level of OATP1A4 and the AUC of Glycyrrhetic acid (*p*<0.01)
